# Shy is a proteobacterial steroid hydratase which catalyzes steroid side chain degradation without requiring a catalytically inert partner domain

**DOI:** 10.1016/j.jbc.2024.107509

**Published:** 2024-06-27

**Authors:** Kurt L. Schroeter, Nicolas Rolfe, Taylor J.B. Forrester, Matthew S. Kimber, Stephen Y.K. Seah

**Affiliations:** Department of Molecular and Cellular Biology, University of Guelph, Guelph, Ontario, Canada

**Keywords:** steroid, hydratase, MaoC, proteobacteria, bile acids, substrate specificity

## Abstract

Shy (side chain hydratase) and Sal (side chain aldolase), are involved in successive reactions in the pathway of bile acid side chain catabolism in Proteobacteria. Untagged Shy copurified with His-tagged Sal indicating that the two enzymes form a complex. Shy contains a MaoC and a DUF35 domain. When coexpressed with Sal, the DUF35 domain but not the MaoC domain of Shy was observed to copurify with Sal, indicating Sal interacts with Shy through its DUF35 domain. The MaoC domain of Shy (Shy_MaoC_) remained catalytically viable and could hydrate cholyl-enoyl-CoA with similar catalytic efficiency as in the Shy-Sal complex. Sal expressed with the DUF35 domain of Shy (Sal-Shy_DUF35_) was similarly competent for the retro-aldol cleavage of cholyl-3-OH-CoA. Shy_MaoC_ showed a preference for C_5_ side chain bile acid substrates, exhibiting low activity toward C_3_ side chain substrates. The Shy_MaoC_ structure was determined by X-ray crystallography, showing a hot dog fold with a short central helix surrounded by a twisted antiparallel β-sheet. Modeling and mutagenesis studies suggest that the bile acid substrate occupies the large open cleft formed by the truncated central helix and repositioning of the active site housing. Shy_MaoC_ therefore contains two substrate binding sites per homodimer, making it distinct from previously characterized MaoC steroid hydratases that are (pseudo) heterodimers with one substrate binding site per dimer. The characterization of Shy provides insight into how MaoC family hydratases have adapted to accommodate large polycyclic substrates that can facilitate future engineering of these enzymes to produce novel steroid pharmaceuticals.

Steroids are a structurally and functionally diverse class of terpenoid lipids produced by eukaryotes and, in a very few instances, also by prokaryotes (1). Steroids such as cholesterol, β-sitosterol, and ergosterol make up a significant portion of cell membranes in animal, plant, and fungal cells, respectively (2). Bile acids are hydroxylated amphipathic cholesterol derivatives that are synthesized in the liver of animals and secreted into the digestive tract to emulsify dietary fats, facilitating absorption in the intestine (3). These natural steroids are eventually deposited into the environment and represent a significant potential carbon source. Despite their ubiquity in many environments (4, 5), steroids are inaccessible to most organisms due to their complex structure (including two quaternary carbon atoms and limited functional groups) rendering them resistant to degradation. The ability to utilize steroids as a sole carbon and energy source for growth is limited to certain bacteria from the Proteobacteria and Actinobacteria phyla (6). This rare ability to completely degrade steroids allows these bacteria to fulfill an important role in natural carbon cycling. Metagenomic analysis indicates that these organisms are globally distributed in soil, plant rhizospheres, wastewater treatment plants, and marine environments (6). Furthermore, these bacteria have been harnessed to produce valuable precursors of steroid pharmaceuticals from low-cost phytosterols (7, 8). There are also potential biotechnological applications for the bioremediation of natural and synthetic steroid pollutants in wastewater which commonly originate from livestock farming (9, 10, 11); These pollutants include anthropogenic steroids that can have detrimental effects on the endocrine systems of animals (7, 8). Realizing this wealth of potential applications, however, is hampered by a lack of knowledge of the biochemical details of bacterial steroid degradation pathways and the enzymes involved.

Steroids are comprised of a four-ring nucleus (designated as rings A, B, C, and D) with an alkyl side chain on the D ring. Variations in the length of the D-ring side chain and the placement of hydroxyl substituents on the steroid rings contribute to the structural and therefore functional diversity of steroids (8). The catabolic pathways utilized by the bacteria reflect the structure of these molecules, with distinct pathways responsible for catabolism of the AB-rings, CD-rings, and D-ring side chain. While the pathways of steroid rings catabolism are generally conserved between Actinobacteria and Proteobacteria, the D-ring side chain catabolism pathway is divergent between these phyla. The catabolism of the D-ring side chain is analogous to fatty acid β-oxidation with repeating cycles of enzymatic reactions removing either two carbon atoms as acetyl-CoA, or three carbon atoms as propionyl-CoA (12, 13) (Fig. 1*A*). In Actinobacteria, after activation of the C_5_ carboxylate side chain of cholate with coenzyme A, the resulting thioester can be dehydrogenated by an acyl-CoA dehydrogenase, followed by the addition of water across this double bond by an enoyl-CoA hydratase (ECH) (14, 15). This hydrated product is oxidized by a hydroxyacyl-CoA dehydrogenase to produce a 3-ketoacyl-CoA that can then be cleaved by a β-ketothiolase to form acetyl-CoA (in the case of cholate) to generate a CoA-ester shortened by two carbon atoms (16, 17). This ester then proceeds through an analogous series of reaction steps; however, hydration now results in a tertiary alcohol that cannot be oxidized further and is instead cleaved by an aldolase, Ltp2, releasing propionyl-CoA and a 17-keto steroid (18, 19, 20).

**Figure 1:**
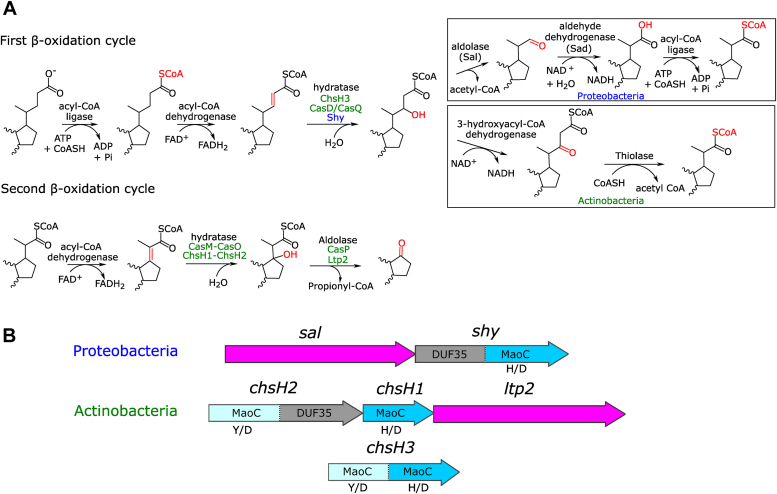
**Overview of steroid side chain catabolism in Actinobacteria and Proteobacteria.***A*, overview of steroid side chain catabolism pathway. Names of relevant enzymes that catalyze the shared steps are indicated in *green* (actinobacterial) and *blue* (proteobacterial). For simplicity the steroid rings are not shown in the figure. *B*, gene arrangement of *shy* and *sal* in Proteobacteria and *chsH1*, *chsH2*, *ltp2,* and *chsH3* in Actinobacteria. The same colors are used to depict the corresponding homologous genes.

Proteobacterial cholate side chain catabolism has been primarily studied in *Stutzerimonas stutzeri* strain Chol1 (previously *Pseudomonas* sp. strain Chol1 (21)), through gene KOs (22, 23). The C-C bond cleavage of the C_5_ side chain of cholate in the first cycle of β-oxidation terminates *via* an aldolytic deacetylation instead of a thiolase catalyzed reverse Claisen reaction (Fig. 1*A*). This aldolytic reaction is thought to be catalyzed by the enzyme Sal (steroid aldolase), producing a free aldehyde which is then oxidized and reesterified to produce a C_3_ CoA-ester which can enter the second round of β-oxidation analogous to the pathway in Actinobacteria (Fig. 1*A*) (22). The two aldolases in the pathway, Sal and Ltp2, are homologous.

Ltp2 was found to associate with the heteromeric hydratase that catalyzes the preceding reaction in the pathway (20). The heteromeric hydratases, exemplified by ChsH1-ChsH2 from *Mycobacterium tuberculosis*, are members of the MaoC family of hydratases. However, only ChsH1 has the canonical MaoC catalytic His/Asp dyad required for the hydration reaction, while the ChsH2 MaoC domain lacks these residues and is modified to accommodate the bulky steroid rings (Fig. 1*B*) (19). Therefore, the steroid rings of the substrate bind to ChsH2 while the side chain extends toward ChsH1 for hydration to occur (19). ChsH2 also possess a DUF35 domain (domain of unknown function 35) at the C-terminus (19) which interacts with the aldolase Ltp2, forming a ChsH1-ChsH2-Ltp2 complex (20). The crystal structure of Ltp2 with the DUF35 domain of ChsH2 showed two DUF35 protein chains at the periphery of a central Ltp2 dimer, bridging the two protomers (24). The DUF35 domain does not appear to be important for the activity of the aldolase or the hydratase (19, 20). The aldolytic cleavage of the hydratase product by Ltp2 is, however, necessary for the hydratase to overcome the unfavorable hydration equilibrium, explaining why the tight coupling of these two enzymes is likely metabolically desirable (20).

Similar hydratases containing two distinct MaoC domains catalyze hydration of the C_5_ steroid side chains in Actinobacteria, including ChsH3 involved in cholesterol catabolism, and CasD and CasQ involved in bile acid catabolism (16, 25). Here, however, the two MaoC domains are fused into a single polypeptide and there are no DUF35 domains (Fig. 1*B*) (25). In contrast, the hydratase, Shy, (side chain hydratase) thought to be responsible for a similar reaction in Proteobacteria, contains a single MaoC domain possessing the His/Asp catalytic dyad in the C terminus and a DUF35 domain in the N terminus (Fig. 1*B*) (23). The *shy* gene is downstream of *sal* and there are no other genes encoding MaoC domains in its vicinity. Since a noncatalytic MaoC domain is seemingly a requirement for the heteromeric hydratases or double MaoC domain hydratases in Actinobacteria to utilize steroid substrates, it is unclear how Shy (which only contains a single MaoC domain) accommodates bile acid substrates. As the function of Shy and Sal are inferred only from gene KO studies, we sought to confirm the function of both Shy and Sal *in vitro*, determine if these enzymes form a complex and whether this association is necessary for the function of each enzyme. We also report the crystal structure of MaoC domain of Shy, shedding light on the molecular basis for steroid binding in this unique homomeric MaoC hydratase.

## Results

### Shy associates with Sal *via* its DUF35 domain

**Figure 2:**
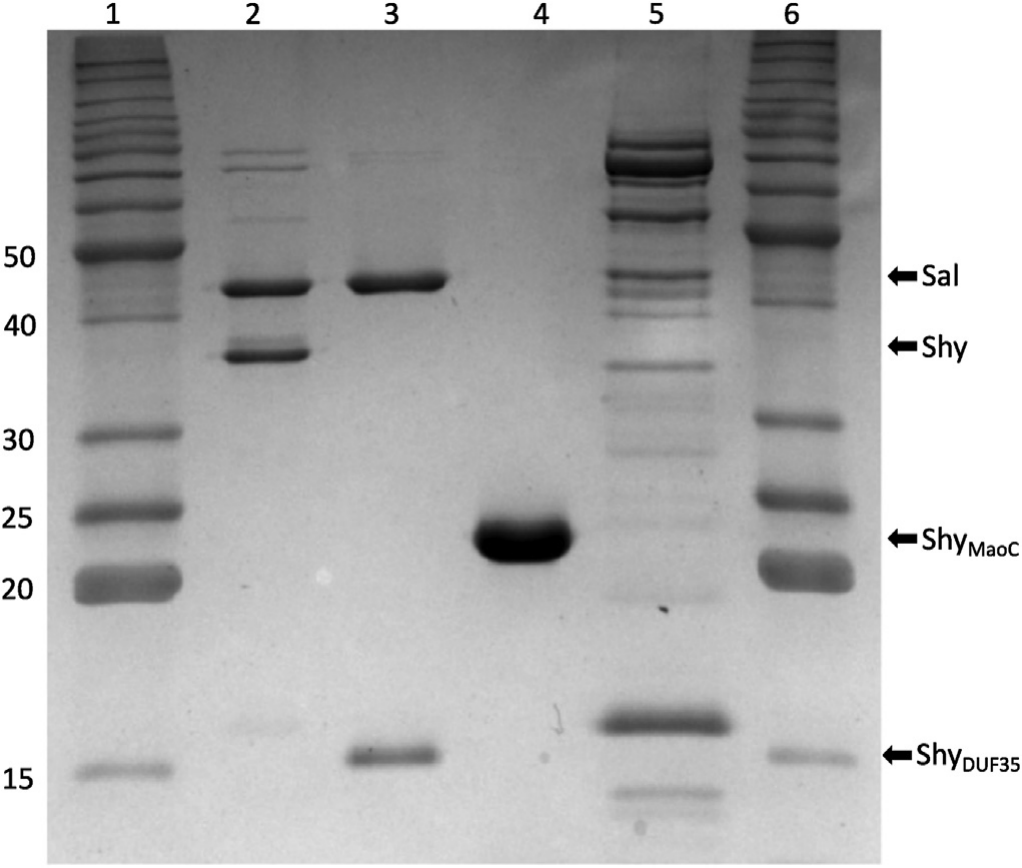
**Coomassie blue-stained 10% SDS-PAGE gel of copurified proteins from Ni**^**2+**^**-NTA chromatography.** Loaded samples are purified from *Escherichia coli* BL21 LOBSTR expressing His-tagged Sal and untagged Shy (lane 2), His-tagged Sal and untagged Shy_DUF35_ (lane 3), His-tagged Shy_MaoC_ (lane 4), and His-tagged Sal and untagged Shy_MaoC_ (lane 5), Along with molecular weight ladders (lanes 1 and 6). Molecular weight of relevant ladder bands in kDa are indicated on the *left* while the positions of the expressed proteins is indicated on the *right*.

Genes encoding Shy and Sal (WP_003057309.1 and WP_003057311.1, respectively) from the Betaproteobacterium *Comamonas testosteroni* KF-1 (23) were amplified from genomic DNA *via* PCR and inserted into the expression vectors. When produced recombinantly and separately in *Escherichia coli*, His-tagged Shy and His-tagged Sal were expressed in low yields and the enzymes were impure after Ni-NTA chromatography. However, when coexpressed, the untagged Shy copurifies with N-terminal His-tagged Sal in good yield on a Ni-NTA column (2.7 mg per liter of culture), indicating that the two enzymes associate (Fig. 2). Purified Shy-Sal, when subjected to analytical size-exclusion chromatography eluted as a single peak corresponding to a molecular weight of 160 kDa, suggesting a stoichiometry of two molecules of Shy and two molecules of Sal in the complex (the theoretical molecular weights of Shy and tagged Sal are 34.81 and 46.08 kDa, respectively) (Fig. S1). We next created a truncated form of Shy that contains the MaoC but not the DUF35 domain (Shy_MaoC_). Shy_MaoC_ can be expressed and purified in good yield from recombinant *E. coli* without Sal. However, untagged Shy_MaoC_ did not copurify with His-tagged Sal when coexpressed. Conversely, an untagged truncated form of Shy containing only the DUF35 domain (Shy_DUF35_) copurified with His-tagged Sal, confirming that Sal interacts with Shy through its DUF35 domain (22).

### Shy-Sal catalyzes hydration and retro-aldol cleavage of cholate substrates

**Figure 3:**
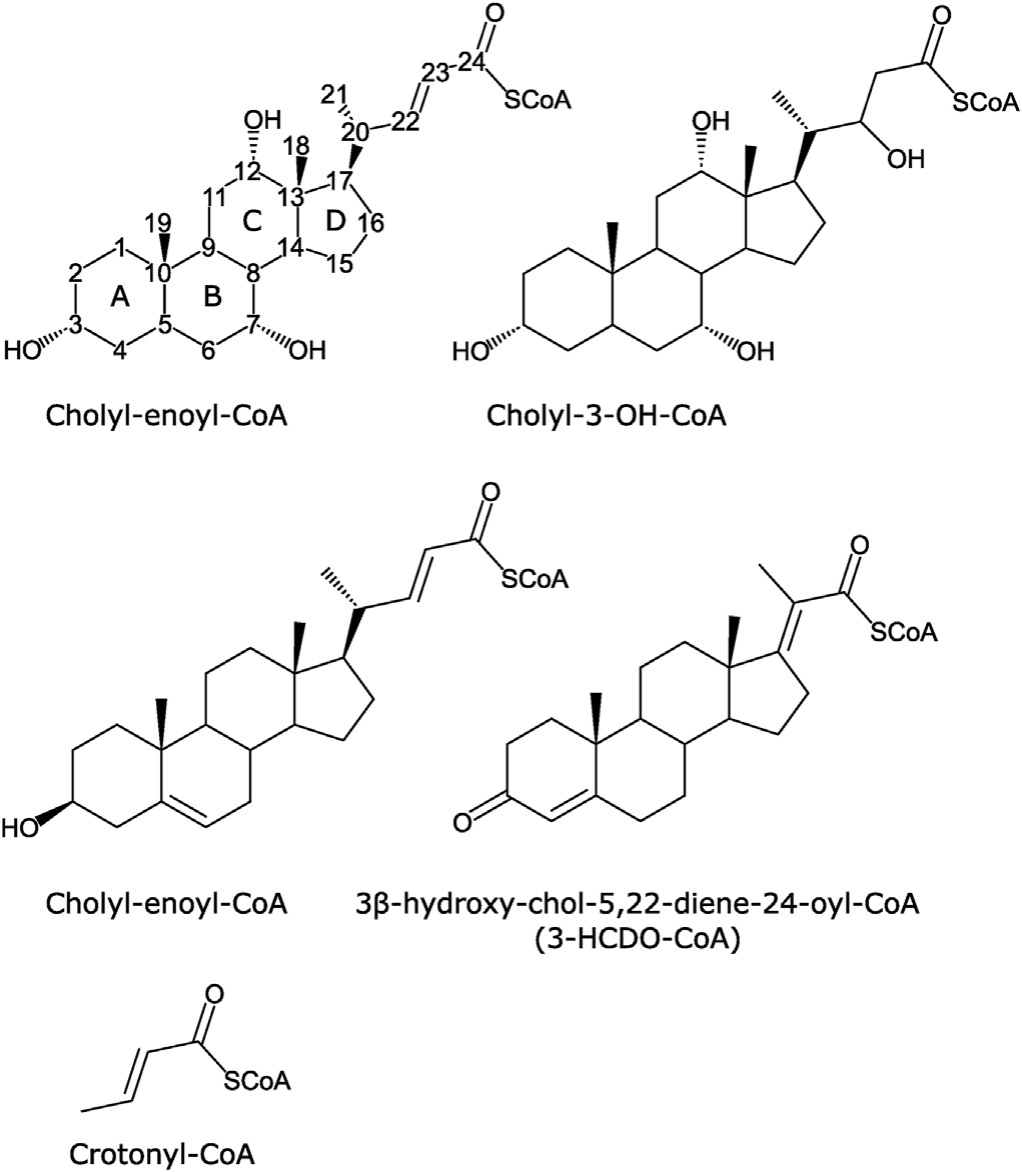
**Structure of synthesized steroid substrates.** Carbon atom numbering scheme and ring designations are shown for Cholyl-enoyl-CoA.

When Shy-Sal was incubated with cholyl-enoyl-CoA (Fig. 3), products corresponding to acetyl-CoA (808.1225 *m/z* [M-H]^−^) and 3,7,12-α-hydroxy-23,24-bisnor-5β-chol-22-al (409.2628 *m/z* [M+HCOO]^−^) were detected *via* LC-MS. When Shy_MaoC_ was incubated with cholyl-enoyl-CoA, the hydrated product cholyl-3-OH-CoA (587.6954 *m/z* [M+2H]^+2^) was detected *via* LC-MS, but no acetyl-CoA or 3,7,12-α-hydroxy-23,24-bisnor-5β-chol-22-al was produced.

The Shy-Sal complex was able to hydrate cholyl-enoyl-CoA with a high catalytic efficiency (*k*_*cat*_*/K*_*m*_) of (2.64 ± 0.191) × 10^7^ M^−1^s^−1^ (Table 1). A similar catalytic efficiency toward the compound was also observed for Shy_MaoC_ indicating that efficient hydration does not require the DUF35 domain nor the association with Sal.

**Table 1:** Steady-state kinetic parameters of Shy and Sal

Reaction	Enzyme	*K*_*m*_ (μM)	*k*_*cat*_ (s^−1^)	*k*_*cat*_*/K*_*m*_ (M^−1^s^−1^)
Hydration of Cholyl-enoyl-CoA	Shy-Sal	6.19 ± 0.446	163 ± 1.50	(2.64 ± 0.191) × 10^7^
	Shy_MaoC_	6.14 ± 0.524	211 ± 6.71	(3.44 ± 0.313) × 10^7^
	Shy_MaoC_I309 F	12.3 ± 1.05	67.2 ± 2.77	(5.46 ± 0.518) × 10^6^
Hydration of 3-HCDO-CoA	Shy_MaoC_	6.71 ± 0.588	62.9 ± 2.32	(9.37 ± 0.89) × 10^6^
Hydration of crotonyl-CoA	Shy_MaoC_	63.6 ± 3.62	(4.12 ± 0.110) × 10^−3^	64.8 ± 4.07
	Shy_MaoC_I309 F	65.0 ± 3.25	(5.55 ± 0.129) × 10^−3^	85.4 ± 4.71
Retro-aldol cleavage of Cholyl-3-OH-CoA	Shy-Sal	8.12 ± 0.477	37.6 ± 0.8	(4.63 ± 0.289) × 10^6^

Specific activity measurements were performed at least in triplicate.

Using a coupled assay where the retro-aldol cleavage of cholyl-3-OH-CoA (Fig. 3) to 3,7,12-α-hydroxy-23,24-bisnor-5β-chol-22-al is linked to reduction of NAD^+^ to NADH by the steroid aldehyde dehydrogenase, Sad from *C. tesosteroni* (23) (Fig. S2), Shy-Sal was found to have a catalytic efficiency of (4.63 ± 0.289) × 10^6^ M^−1^s^−1^ (Table 1). Sal-Shy_DUF35_ was also able to catalyze the retro-aldol cleavage of the compound and LC-MS analysis indicated that Sal-Shy_DUF35_ produced the expected products acetyl-CoA and 3,7,12-α-hydroxy-23,24-bisnor-5β-chol-22-al from cholyl-3-OH-CoA. We noted that Sal-Shy_DUF35,_ however, when diluted, rapidly lost activity. Sal-DUF35_Shy_ has an estimated specific activity of about 8.29 ± 0.11 μmol min^−1^ mg^−1^ using 40 μM cholyl-3-OH-CoA, which is more than two-fold lower in activity compared to the Shy-Sal complex (23.9 ± 0.353 μmol min^−1^ mg^−1^).

We further elucidated the specificity of Shy toward steroid substrates with different side chain lengths and nucleus substituents (Fig. 3). Shy_MaoC_ is capable of hydrating 3β-hydroxy-chol-5,22-diene-24-oyl-CoA (3-HCDO-CoA), which bears a C_5_ side chain and a cholesterol like nucleus, with ∼37-fold decreased catalytic efficiency *versus* cholyl-enoyl-CoA. Shy_MaoC_ possessed low activity toward the C_3_ side chain metabolite 3-oxo-4,17-pregnadiene-20-carboxyl-CoA (3-OPDC-CoA) with a specific activity at 35 μM 3-OPDC-CoA approximately 1000-fold lower than that obtained with the same concentration of cholyl-enoyl-CoA ((3.70 ± 0.305) × 10^−2^ *versus* 51.2 ± 0.0833); this activity was too low to determine kinetic parameters. Given that 3-OPDC-CoA has a similar nucleus to 3-HCDO-CoA, these results suggest Shy is specific toward C_5_ side chains and has a broad specificity for steroid ring nucleus conformations and substituents, with a preference for bile acid nuclei. Lastly, Shy_MaoC_ was assayed for activity against crotonyl-CoA, an aliphatic enoyl-CoA with C_4_ acyl chain. The catalytic efficiency for this substrate is six orders of magnitude lower than that for cholyl-enoyl-CoA, indicating that the steroid ring nucleus is an important determinant for productive substrate binding.

### Structure of Shy_MaoC_

**Figure 4:**
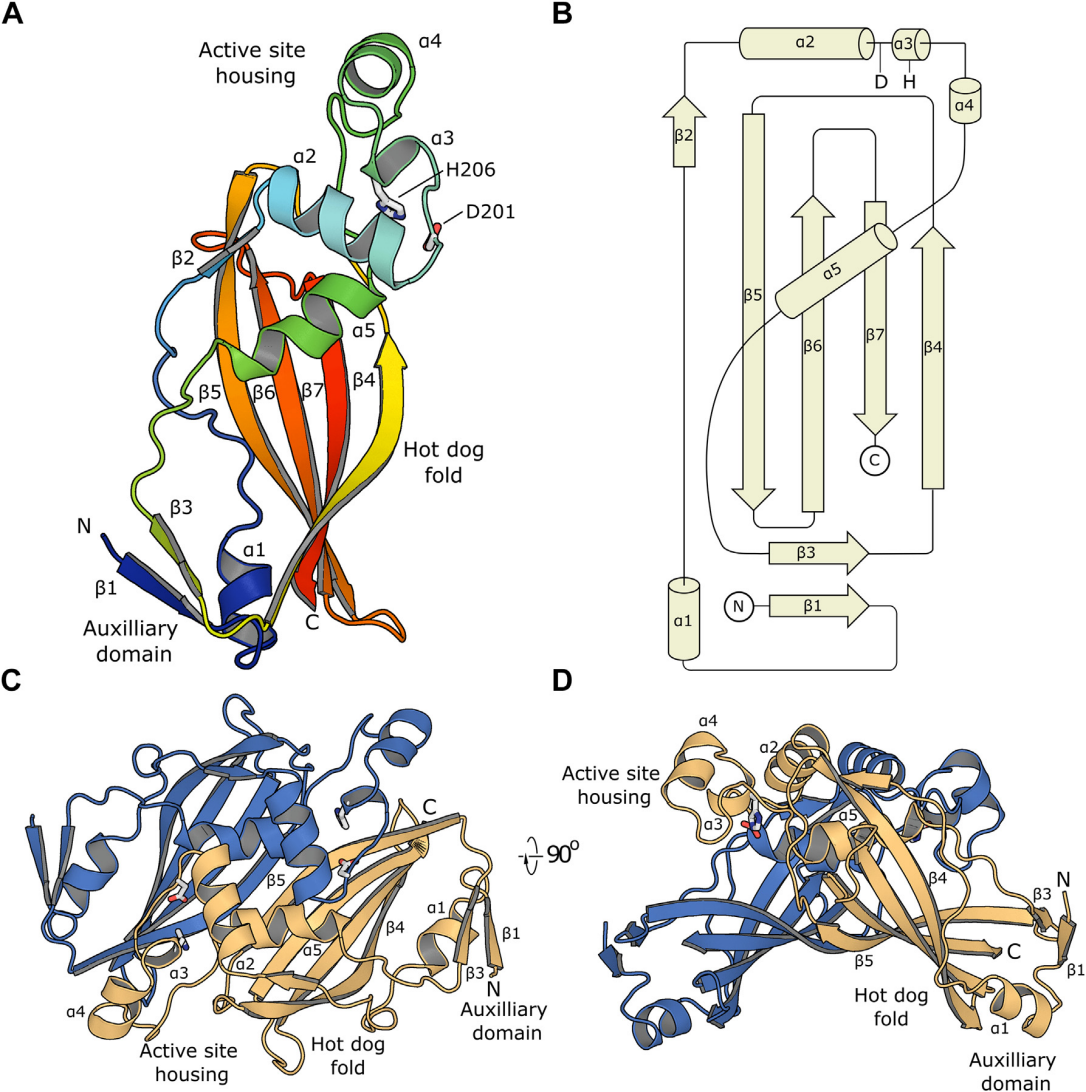
**Structure of Shy**_**MaoC**_**.***A*, secondary structure organization of Shy_MaoC_ with secondary structure elements labeled. Side chains of the catalytic His and Asp residues are shown in *sticks*. *B*, topology diagram of Shy_MaoC_. Approximate positions of the catalytic His and Asp residues are indicated. *C*, organization of Shy_MaoC_ homodimer with protomers colored *blue* and *orange*. Key secondary structure elements for the *orange* protomer are labeled. *D*, Shy_MaoC_ homodimer depiction rotated 90° from (*C*).

The crystal structure of Shy_MaoC_ was determined to 2.05 Å resolution by molecular replacement using a protomeric model of the protein as predicted by ColabFold (26) as the search model. Data collection and structure refinement statistics are summarized in Table 2; electron density is shown in Fig. S3. Although the crystal was grown in the presence of acetyl CoA, electron density for the ligand was not observed in the structure. Shy_MaoC_ crystallizes with two chains in the asymmetric unit; these protomers superimpose closely with an r.m.sd of 0.3 Å. In both protomers, the first 13 residues (E_148_SEVAVVEQSVAA_158_) are disordered and were not modeled. Shy_MaoC_ adopts a hot dog fold, with a central bent five-stranded antiparallel β-sheet (topology 2, −5, 6, −7, 4) “bun” wrapping around a central helix “sausage”, inserted between β2 and β3 (Fig. 4, *A* and *B*). A three-helix motif that contains residues important for catalysis is inserted N terminal to the central helix; this motif is termed the active site housing segment (27, 28). An additional auxiliary domain is built from the N-terminal strand-helix pair β1 and α1, with β1 paired parallel with β3 (contributed by the loop joining the central helix, α5, to β4); this auxiliary domain is positioned at the end of the central β-sheet distal from the active site housing. The two Shy_MaoC_ protomers interact to form a homodimer with an interface area of 1001.9 Å^2^ (as determined by proteins, interfaces, structures and assemblies, PISA) (Fig. 4*C*) (29). β4 strands from adjacent protomers interact in antiparallel fashion to form an extended 10-strand β-sheet; in addition, the central helix, α5, and α4 from the active site housing of each protomer interact to form a four-helix bundle which buries an extended nonpolar surface. Analytical size-exclusion chromatogram of Shy_MaoC_ displays a single peak corresponding to a molecular weight of 40 kDa, suggesting that a homodimer is the preferred oligomeric state of this construct in solution (the theoretical molecular weight of tagged Shy_MaoC_ is 20.5 kDa) (Fig. S1).

**Table 2:** Data collection and refinement statistics

Data collection	
Space group	P3_2_21
Cell dimensions	
*a, b, c* (Å)	95.28, 95.28, 98.59
α, β, γ (°)	90, 90, 120
Resolution range (Å)	47.64–2.05 (2.123–2.05)
Reflections	
Total reflections	657,324 (68,203)
No. of unique reflections	32,918 (3235)
*R*_*sym*_	0.1249 (3.193)
*R*_*meas*_	0.1282 (3.272)
*R-pim*	0.02896 (0.7095)
*CC*_*1/2*_	0.999 (0.492)
*I*/σ*I*	13.81 (1.00)
Completeness (%)	99.96 (99.97)
Multiplicity	20.0 (21.1)
Refinement	
No. of reflections	32,909 (3235)
No. of reflections for *R*_free_	1648 (167)
*R*_work_	0.1971 (0.3277)
*R*_free_	0.2236 (0.3643)
No. atoms	2608
Protein	2452
Ligand/ion	142
Water	96
Wilson *B*-factor (Å^2^)	49.72
*B-*factors (Å^2^)	
Protein	70.66
Ligand/ion	90.77
Water	66.43
RMSD	
Bond lengths (Å)	0.003
Bond angles (°)	0.55
Ramachandran statistics	
Favored (%)	97.04
Allowed (%)	2.96
Outliers (%)	0

∗Data for highest resolution shell shown in parentheses.

### Comparison to other hydratases

**Figure 5:**
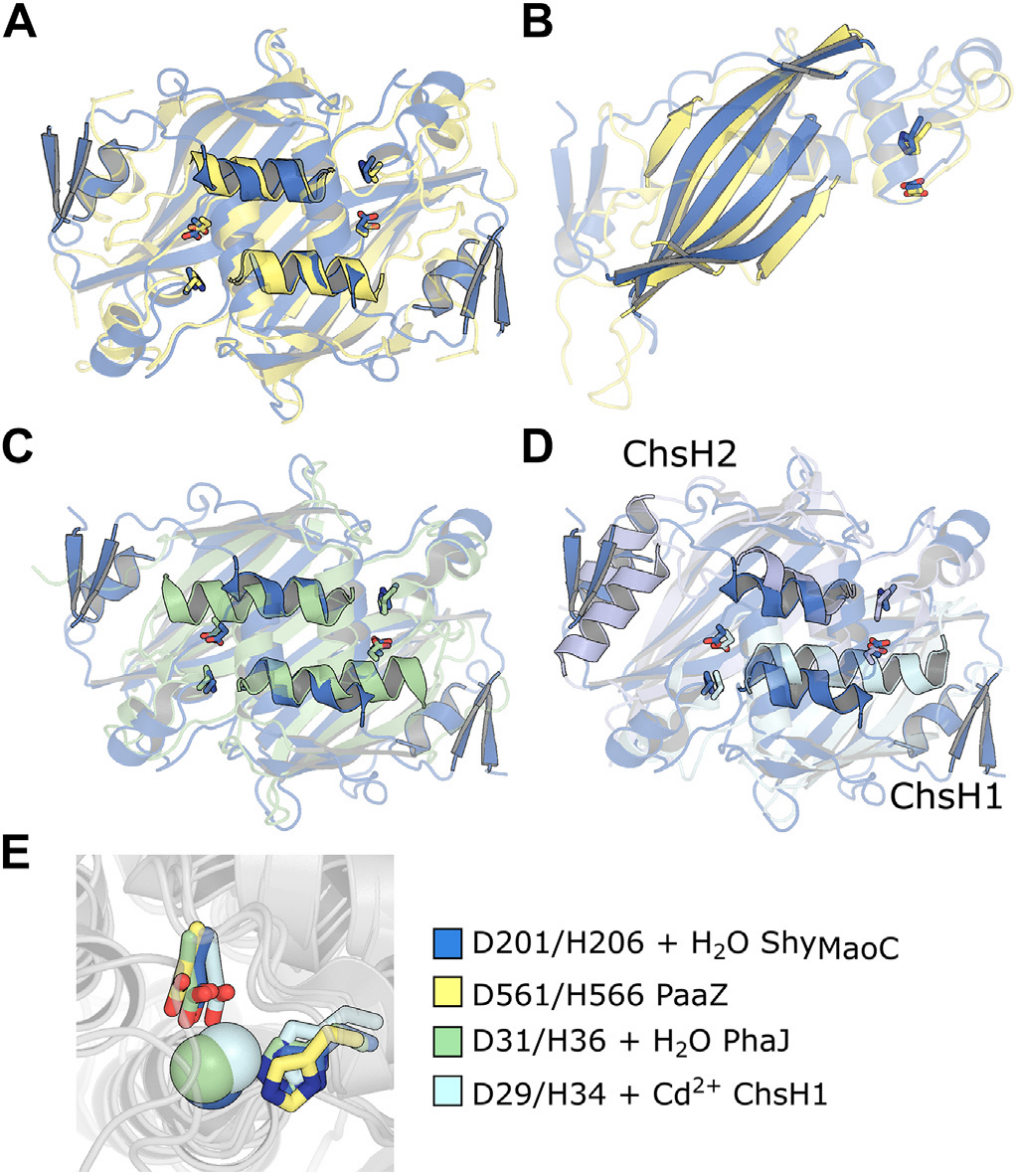
**Comparison of Shy**_**MaoC**_**, PaaZ**_**MaoC**_**, PhaJ**_**Acav**_**, and ChsH1-ChsH2 Structures.***A*, structural alignment of Shy_MaoC_ dimer (shown in *blue*) with PaaZ_MaoC_ dimer (shown in *yellow*), secondary structure elements outside of the central helix are semi-transparent for clarity and the catalytic dyad are shown as sticks. *B*, structural alignment of Shy_MaoC_ protomer with PaaZ_MaoC_ protomer (colored as in *A*), secondary structure elements outside of the central β-sheet are translucent for clarity. *C*, structural alignment of Shy_MaoC_ dimer with PhaJ_Acav_ dimer (shown in *green*), displayed as in *A*. *D*, structural alignment of Shy_MaoC_ dimer with ChsH1-ChsH2 dimer (shown in *cyan* and *lavender*, respectively), displayed as in (*A*). *E*, structural alignment of Shy_MaoC_, PaaZ_MaoC_, PhaJ_Acav_, and ChsH1-ChsH2 showing the catalytic dyads, coordinated water of Shy_MaoC_ and PhaJ_Acav_, and coordinated cadmium cation of ChsH1 colored as in (*A*–*C*).

Structural homologues of Shy_MaoC_ were initially identified *via* a heuristic Protein Data Bank (PDB) search using DALI (http://ekhidna2.biocenter.helsinki.fi/dali/) (statistics listed in Table S1) (30). The highest ranking hit that has been biochemically characterized is the C-terminal ECH domain of PaaZ from *E. coli* K12 (PaaZ_MaoC_) (PDB ID: 6JQN), a bifunctional enzyme involved in phenylacetic acid metabolism (31) (Fig. 5*A*). PaaZ_MaoC_ hydrates oxepin-CoA, a seven-membered C-O heterocyclic compound with a C_2_ side chain, with high catalytic efficiency (32). An interesting aspect of this structural similarity is that PaaZ_MaoC_, unlike most other MaoC domains, shares a pronounced twisting of the β-sheet with Shy (Fig. 5*B*). Another high-ranking hit is PhaJ from *Aeromonas caviae* (PhaJ_Acav_) (PDB ID: 1IQ6), an ECH involved in the biosynthesis of polyhydroxyalkanoates (PHAs) and which is specific toward short chain enoyl-CoAs with C_4_-C_6_ acyl chains (27, 33, 34) (Fig. 5*C*). Surprisingly, the steroid specific ChsH1-ChsH2 ECH from *M. tuberculosis* (PDB ID: 4W78), while also among the high-ranking hits, is less similar to Shy than PaaZ and PhaJ despite also utilizing steroid substrates (Fig. 5*D*). ChsH1-ChsH2 is specific toward C_3_ side chain steroid substrates (3-OPDC-CoA) and can also hydrate crotonyl-CoA with a three order of magnitude reduced catalytic efficiency *versus* 3-OPDC-CoA (19, 25).

The three-helix active site housing motifs in these proteins are structurally conserved and, except for the noncatalytic ChsH2, contain the conserved catalytic dyad comprised of a water activating histidine (substituted with tyrosine in ChsH2) and an aspartate that positions the water for hydration (Fig. 5*E*). The catalytic roles of these residues have been well established in multiple enzymes including ChsH1 and PhaJ_Acav_ (19, 27, 35). The water molecule that hydrates the substrate in PhaJ_Acav_ is observed in an equivalent position in Shy_MaoC_ within hydrogen bond distance to the carboxylate of Asp-29, while in ChsH1 the catalytic water is displaced by a cadmium ion (28).

All of these proteins form dimers with a similar interface, including the pairing of each protomer’s β-sheet into an extended 10-strand antiparallel β-sheet and packing between the adjacent active site housings. Shy_MaoC_, PhaJ_Acav_, and PaaZ_MaoC_ form homodimers with two active sites at the dimer interface, in which the active site housing from one residue faces a substrate-binding pocket formed by the bun and helix of the opposite protomer. In contrast, the ChsH1-ChsH2 heterodimer contains only one active site made up of the active site housing of ChsH1 and the substrate binding pocket of ChsH2.

### Active site architecture of Shy

**Figure 6:**
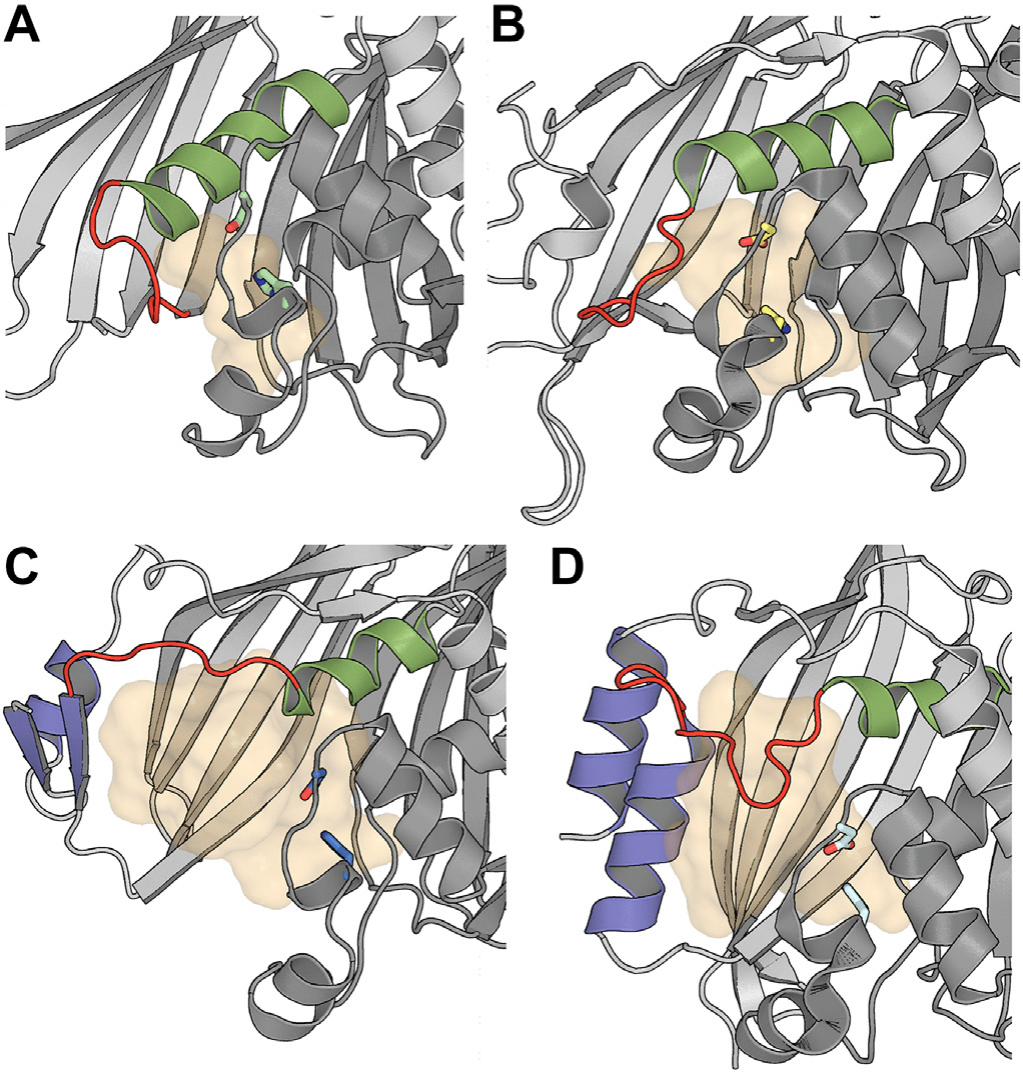
**Comparison of PhaJ**_**Acav**_**, PaaZ**_**MaoC**_**, ChsH1-ChsH2, and Shy**_**MaoC**_**substrate binding pockets.***A*, PhaJ_Acav_, (*B*) PaaZ_MaoC,_ (*C*) Shy_MaoC_, and (*D*) ChsH1-ChsH2. Each dimer shows a single substrate binding site in *light orange*. The protomer contributing the catalytic dyad is *dark gray*, with the residues shown as *sticks*. The opposite protomer is *light gray*, with the central helix and connecting loop that define the end of the binding site colored *green* and *red*, respectively. In ChsH1-ChsH2 and Shy_MaoC_ the auxiliary domain is colored *purple*. Pockets were detected using HOLLOW (58) and their volumes were estimated with 3V (59).

In canonical MaoC ECHs like PhaJ the active site takes the form of an enclosed tunnel with a single, narrow opening through which the acyl portion of the substrate is threaded. The depth of the PhaJ_Acav_ tunnel is defined in part by the central helix “sausage”, which is 16 residues in length and connected to the equivalent of β4 through a short loop. The tunnel entrance is defined by the active site housing of the opposite protomer which contacts the central helix, connecting loop, and β-strand (Fig. S4*A*). The long central helix of PhaJ restricts the volume of the central substrate-binding cavity, resulting in a relatively short tunnel with an approximate volume of 827 Å^3^ (Fig. 6*A*). PaaZ_MaoC_ possesses a similar enclosed tunnel like active site; (Fig. S4*B*). However, the central helix is shortened by three residues at its C-terminal end, with a longer loop connecting it back to the subsequent β-strand. The shortened helix increases the volume of the tunnel to approximately 1144 Å^3^, consistent with the larger physiological substrate of PaaZ_MaoC_ (Fig. 6*B*).

In contrast, Shy_MaoC_ represents a remodeling of this canonical MaoC active site architecture. Firstly, the central helix of Shy_MaoC_ is only nine residues long, with the shortening of the helix by an additional turn at the C terminal (relative to the already shortened PaaZ_MaoC_) opening up considerable additional space in the active site. This helix also shows mixed geometry, with the first four hydrogen bonds conforming to an α-helical pattern (i to i + 4), while the subsequent four follow a 3_10_-helix pattern (i to i + 3); this narrows the C-terminal half of this helix. The loop C terminal to this central helix is also greatly elongated, forming β3 of the auxiliary domain before linking back to β4 of the central β-sheet; this pushes these residues to the periphery of the binding site, where they form an extended wall of an open pocket rather than forming one side of the substrate binding tunnel. In order to accommodate this loop, the N-terminal end of the otherwise long (in PhaJ_Acav_ and PaaZ_MaoC_) β2 strand does not hydrogen bond with the central β-sheet, instead packing behind it as an extended loop. Together, these changes expose much of the surface of the β-sheet to solvent. The active site is further opened up by a small shift outward of the active site housing; as a result of this shift (along with the shortening of the central helix and the α5-β3 loop) α3 and α4 do not make contact with the dimeric partner (Fig. S4*C*). Together these changes produce a continuous open cleft that spans the width of the β-sheet with an approximate volume of 2895 Å^3^ (Fig. 6*C*).

While the single active site of ChsH1-ChsH2 also diverges from the canonical MaoC organization, the remodeling is less extensive than observed in Shy_MaoC_. Specifically, the central helix of ChsH2 is also nine residues long with mixed geometry but with the N terminal most four residues following a 3_10_ pattern, while the final three residues are α-helical. ChsH2 also features an auxiliary domain, albeit with a different composition than Shy_MaoC_, with the extended loop C-terminal to the central helix connecting to an α-helix which forms a two-helix bundle with an N-terminal helix. Intriguingly, ChsH2 also reorganizes the edge of β-strand 2 of the hot dog “bun” into a loop which connects α1 to the active site housing, analogous to the α1 to β2 loop in Shy_MaoC_. A key difference between ChsH1-ChsH2 and Shy is that the active site housing of ChsH1 maintains contact with the β-sheet maintaining the canonical tunnel entrance through which CoA is threaded, but with an extended “back door” opening formed by the remodeling of the ChsH2 active site (Fig. S4*D*). The distinct auxiliary domain of ChsH2 is positioned closer to the dimer core relative to Shy_MaoC_, which combined with the tunnel entrance results in a reduced internal volume of approximately 2017 Å^3^ (Fig. 6*D*). As previously mentioned ChsH2 lacks the catalytic histidine required for activation of water for hydration, in addition, the substrate-binding pocket formed by ChsH1 is too small to accommodate steroid substrates due to the 18 residue long central helix that connects directly back to the central β-sheet *via* a short loop without an intervening auxiliary domain, forming a restrictive enclosed tunnel akin to canonical MaoC ECHs. Overall, the architecture of the active site of ChsH1-ChsH2 differs significantly from Shy_MaoC_ despite both hydratases using steroidal substrates.

We next constructed a maximum likelihood phylogenetic tree from these three types of hydratases (Fig. S5). Homologs of PaaZ and PhaJ were selected, along with Shy homologous from proteobacteria confirmed to catabolize steroids. Homologs of ChsH1 and ChsH2 were selected from cholesterol degrading actinobacteria while their homologs, CasM and CasO, were selected from bile acid degrading actinobacteria. To align the MaoC domains of these proteins the DUF35 domains and the aldehyde dehydrogenase domains of all sequences were removed from the alignment. The resulting tree shows that Shy is most closely related to the other proteobacterial enzymes, sharing, instead, a most recent common ancestor with PaaZ followed by PhaJ. The heteromeric hydratases form distinct clades, with catalytic ChsH1/CasO and noncatalytic ChsH2/CasM grouping together.

### Determinants of steroid specificity

**Figure 7:**
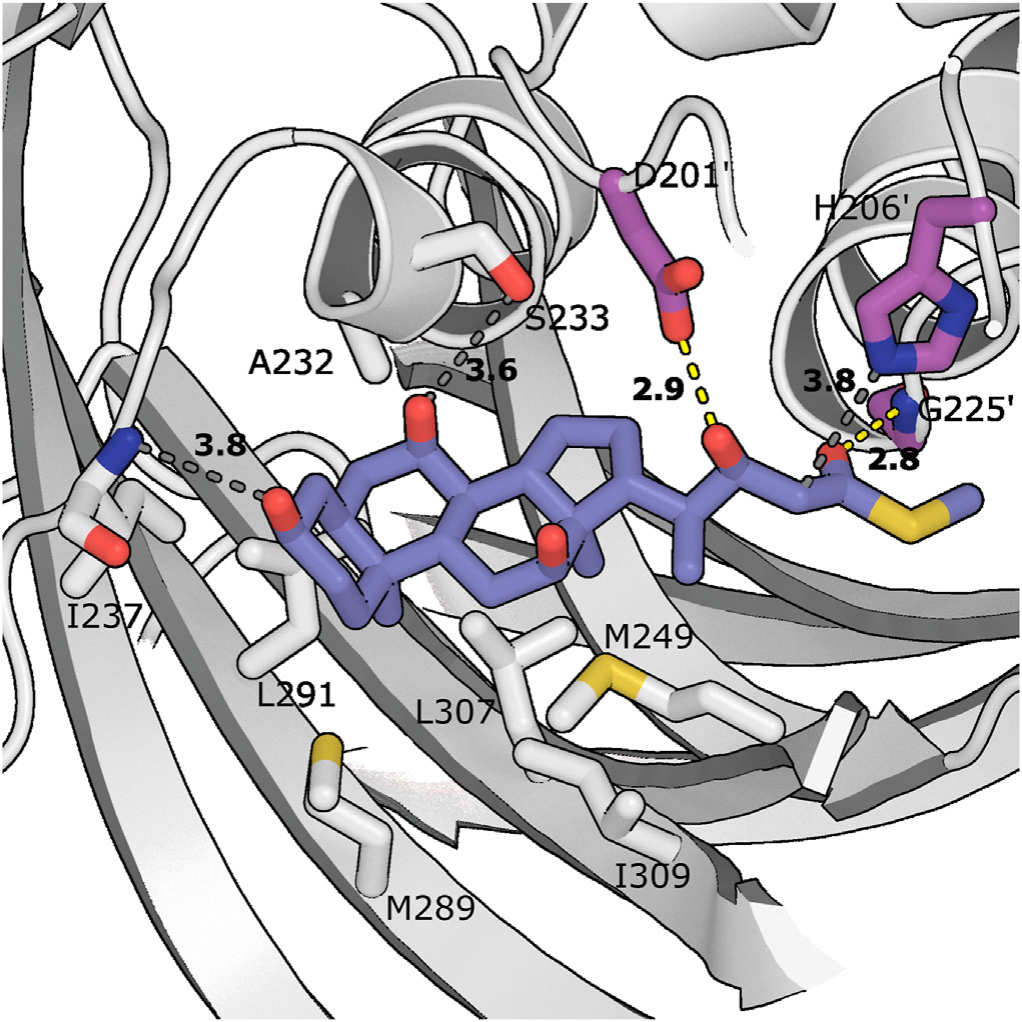
**Structure of Shy**_**MaoC**_**substrate binding site with modeled cholyl-22-(*R*)-hydroxy-24-methylthioate.** Shy_MaoC_ is shown in *gray* while the substrate is shown in *purple*. The functional groups of residues that define the binding cleft and participate in catalysis are shown in *gray* and *magenta*, respectively, and numbered (' indicates residues from the opposite protomer). Potential hydrogen bonds are shown as *dashed lines* with their length in angstroms indicated. Those between the substrate and catalytic residues are colored *yellow*, while those between the substrate and noncatalytic residues are *gray*.

To gain further insight on steroid binding, a derivative of cholyl-3-OH-CoA (cholyl-22-(*R*)-hydroxy-24-methylthioate), the product of the hydration of cholyl-enoyl-CoA, was modeled into the structure of Shy_MaoC_ and energy minimized using Rosetta (Fig. 7) (36). Based on the consistent stereospecificity of other MaoC hydratases the 3-hydroxyl group was placed in the *R*-configuration. CoA is both large and highly flexible; the docking ligand was therefore terminated as a methyl thioester so that interactions with the relatively rigid, nonpolar steroid moiety drive binding. The resulting structure shows extensive ligand-protein interactions, with both good nonpolar shape complementarity and formation of favorable hydrogen bonds, including those required for catalysis (35). In the forward direction, MaoC hydratases catalyze the syn addition of water to *trans*-α,β double bonds, protonating the α carbon and hydroxylating the β carbon from the same side (35). The water molecule is positioned *via* hydrogen bonding with the catalytic Asp and His, with the His acting as the catalytic base/acid, deprotonating the water which then attacks the β-carbon, followed by protonation of carbon α (Fig. S6) (35). The third conserved catalytic residue is a Gly located on the N-terminal end of the central helix, the backbone amide of this residue hydrogen bonds to the thioester carbonyl oxygen of the substrate, forming an oxyanion hole that acts to stabilize the enolate intermediate formed by the attack on the β-carbon (35). The modeled substrate exhibits all the expected interactions required for the reverse reaction; the 3-hydroxyl group is positioned to hydrogen bond with Asp-201, with His-206 positioned in range to act as the catalytic base, deprotonating the α-carbon (35). Cα-Cβ of the substrate are positioned such that the resulting enoyl double bond would be trans, and the carbonyl oxygen is positioned to hydrogen bond with the amide of Gly-225, forming the conserved oxyanion hole interaction.

The steroid nucleus packs across the β-sheet, interacting with an extended hydrophobic patch comprised of the side chains of Met-289 and Leu-291 on β5, Leu-307 and Ile-309 on β6, and Met-249 on β4. The distal edge of the pocket shifts inward during minimization, allowing Met-249 to contact the edge of the C-ring. This surface patch shows good shape complementarity with the substrate, with defined pockets for the C18 and C19 methyl carbons. The bent conformation of the bile acid nucleus, induced by the *cis* conformation at the A/B ring juncture, places the A ring out plane with the rest of the nucleus. This ring interacts with residues from the loop connecting the C terminus of the central helix with the auxiliary domain, with the side chain of Ile-237 extending downward and interacting with the β-face of the A ring. The C3-α-hydroxyl is positioned close enough to the exposed amide nitrogen of Ile-237 (3.8 Å) that additional small local adjustments may allow a hydrogen bond to form; alternatively, a water molecule might bridge these groups. The back of the pocket is delimited by Ala-232 and Ser-233 at the C-terminal end of the central helix, with the Ser-233 hydroxyl within 3.6 Å of the 7α-hydroxyl group; the shortening of this helix, and its narrower 3_10_ character in this region, creates the necessary space to accommodate the bulky steroid ring. Note that the steroid ring binds with its hydrophobic β-face interacting with the enzyme, while the hydrophilic α-face remains exposed to the solvent. Because the bile acid substrate is rigid and interfaces with the enzyme primarily through nonpolar interactions, binding appears to be primarily driven by shape complementarity between the steroid ring and the exposed nonpolar surfaces.

The side chain methyl thioester is positioned proximal to the gap between the β-4 strands of the adjacent protomers. This positioning coincides with the CoA binding orientation observed in other hydratases, including PaaZ and ChsH1-ChsH2 where a typical “boomerang” conformation bends the cysteamine arm through the notch between β4 strands and around the β-sheet, while positioning the ADP moiety on the opposite face of the β-sheet. In the structure of PaaZ in complex with octanoyl-CoA, Tyr-607 and Arg-258 from opposite protomers form hydrogen bonds with the 3′ adenine phosphate, while Lys-636 from the same protomer as Arg-258 forms hydrogen bonds with the 5′ β-phosphate (31). Tyr-252 and Arg-232 from opposite protomers in Shy_MaoC_ superimpose well with the corresponding PaaZ residues (Fig. S7*A*). Additionally, sequence conservation analysis in ConSurf (37, 38) of Shy_MaoC_ shows that both Tyr-252 and Arg-232 exhibit strong conservation (Fig. S7*B*). Together this suggests that Tyr-252 and Arg-232 are plausibly involved in CoA binding, and that CoA in Shy_MaoC_ is likely to bind similarly to that observed in PaaZ.

To test the substrate binding model in Shy_MaoC_, we selected residues within the proposed binding site for mutagenesis. In addition, we attempted mutagenesis of the conserved catalytic histidine. We generated Phe replacements of Leu-307 and Ile-309 and Ala and Glu replacements of His-206. Unfortunately, L307F, H206A, and H206Q expressed at low yield and purity, precluding further CD analysis; however, I309F expressed well and had a CD spectrum consistent with WT Shy_MaoC_ (Fig. S8). Steady state kinetic parameters for Shy_MaoC_I309F were acquired with cholyl-enoyl-CoA and crotonyl-CoA. Shy_MaoC_I309F had a two-fold increased *K*_*m*_ for cholyl-enoyl-CoA and an approximately three-fold decreased k_cat_, resulting in an approximately six-fold reduction in catalytic efficiency (Table 1). The model predicts that Ile-309 packs closely on the cholyl-enoyl-CoA C19 methyl group; the increase in bulk and reduction in flexibility introduced by the I309F replacement would be anticipated to introduce steric clashes that result in suboptimal binding and potentially reduce the fraction of time the bound substrate is able to adopt a productive binding orientation. In contrast, this replacement would not impact the binding of the much smaller crotonyl-CoA; consistent with this, the catalytic efficiency toward crotonyl-CoA was nearly identical to the WT enzyme.

## Discussion

The C-C bond cleavage in the first round of β-oxidation of bile acid side chains is catalyzed by an aldolase in Proteobacteria, instead of by a thiolase. We showed that this aldolase, Sal, forms a complex with the hydratase, Shy, that catalyzes the preceding reaction. Shy contains a MaoC domain and a DUF35 domain, the latter which is responsible for interaction with Sal. Notably the DUF35 domain is dispensable for substrate hydration, with the isolated MaoC domain (Shy_MaoC_) retaining the catalytic efficiency of the full protein. This is analogous to the hydratase-aldolase complex ChsH1-ChsH2-Ltp2 involved in the last round of β-oxidation reactions in bacterial steroid degradation where the DUF35 domain involved in hydratase-aldolase association is not essential for the hydration activity of ChsH1-ChsH2. However, Shy differs from the corresponding ChsH1-ChsH2 hydratase, as it is homomeric instead of heteromeric and is specific for C_5_ side chain rather than C_3_ side chain steroid metabolites.

The structure of Shy_MaoC_ reveals a drastically remodeled active site, with a large, exposed cleft resulting from the truncated central helix and extended, repositioned loops; this creates space for the bulky gonane nucleus. The other major change is the opening of the active site entrance *via* repositioning of the active site housing, resulting in the narrow tunnel mouth typical of MaoC hydratases being replaced with an extended open cleft. This suggests that accommodation of the steroid substrate not only requires increasing the size of the substrate binding pocket but also the size of the entrance, as the large rigid steroid substrate may be too unwieldy to thread through a tunnel opening like a linear acyl chain could in an ECH like PhaJ. While the active site housing helices α2 and α3 of ChsH1 do make contact with ChsH2, the contact area is much smaller than seen in PhaJ_Acav_ or PaaZ_MaoC_, and this motif may possibly shift to open up the active site and allow entry of the substrate.

While the shape and size of Shy’s substrate binding site is quite distinct from other MaoC hydratases, the general mode of substrate binding share many similarities to PhaJ_Acav_. Docked structures of PhaJ_Acav_ and other *Pseudomonas* homologues place the acyl tail of the substrate with the ω-carbon interacting with the longer central helix (37, 39). The Shy docked model places the gonane nucleus of the steroid along a similar axis, with the A/B rings extending into the space opened by the shortened central helix, sitting alongside the end of the helix and interacting with the distal connecting loop. The conservation of CoA binding residues from PaaZ_MaoC_ also supports the general position of the substrate being similar to these canonical enzymes. This similarity extends to the specific amino acids side chains involved in substrate binding. In PhaJ_Acav_ and homologs from *Pseudomonas aeruginosa* and *Pseudomonas putida* (37, 39), the width of the active site is defined on one side by a Ser residue on the central helix, while the opposite side is defined by a Val/Ile residue on the central β-sheet. These residues are conserved in Shy_MaoC_ from *C. testosteroni* which has a Ser residue on the central helix and an Ile residue on the respective position on the β-sheet. Shy homologs from other Proteobacteria also contain a conserved Ser and Ile/Met/Val residue at the respective positions (Fig. S9). The modeled structure suggests that the Cδ methyl group of this Ile residue (Ile-309) on β7 of Shy_MaoC_ is in van der Waals contact with C11 ring carbon and the C18 and C19 methyl carbon groups; replacement with the bulkier Phe led to reduced activity with the steroid substrate. The other residues interacting with the steroid rings identified in the docked structure are also conserved in various Shy homologues. Together, this suggests that the adaptations to accommodate the steroid nucleus in Shy require significant remodeling of secondary structure and main chain geometry, where an alternative set of exposed residues define the distal side of the binding pocket, but those closest to the catalytic dyad are conserved.

Shy’s ability to catalyze reactions with steroid substrates without requiring a noncatalytic dimeric partner does call into question the prevalence of a specialized inactive MaoC domain in actinobacterial and eukaryotic steroid hydratases, such as in ChsH1-ChsH2. While ChsH1-ChsH2 and its bile acid degrading homologs are specific toward C_3_ side chains, heteromeric/pseudoheteromeric MaoC ECHs are also involved in β-oxidation of steroids of different structures. For example, Mfe-2 ECH in mammals uses cholesterol metabolites with a C_8_ side chain (38), while CasD and CasQ in actinobacteria use bile acid metabolites with a C_5_ side chain (25). Therefore, the specialized domain is not simply due to constraints for binding steroid substrates of specific side chain lengths or ring structures.

In MaoC hydratases specific toward small substrates, such as PhaJ, the longer central helix spans the entire length of the β-sheet bun, forming extensive interactions that serve to stabilize the protein. When the central helix is shortened to accommodate larger substrates, these interactions are no longer present, requiring other stabilizing interactions to compensate. Homodimeric proteins such as Shy and (pseudo)heterodimeric proteins such as ChsH1-ChsH2 with a specialized domain may represent two distinct solutions to this issue. In heterodimers, the specialized substrate binding subunit with the short helix (ChsH2) may be stabilized *via* association with the catalytic unit (ChsH1). ChsH1 possesses a long helix, closing off its substrate binding pocket, which may serve to brace ChsH2 and compensate for the stabilizing interactions lost due to the short ChsH2 helix. In contrast Shy could be considered a homodimer of ChsH2 analogues, with both proteins having short central helices. The β-sheet of Shy_MaoC_ has a pronounced twist that is especially notable in the distal strand (β5). The twist in these sheets may introduce increased rigidity that stabilizes the sheet *in lieu* of interactions between the helix and sheet, circumventing the need for a binding partner with a longer central helix to stabilize the dimer. A similar twist can be observed in PaaZ_MaoC_, which also accommodates a large cyclic substrate without the need for a specialized domain. In addition, Shy_MaoC_ also features an auxiliary domain which packs against the exposed end of the sheet. Given that the central helix of Shy is even shorter than that of PaaZ_MaoC_, this domain may serve to buttress the β-sheet of Shy, further stabilizing it. ChsH2 has an analogous domain comprised of a two-helix bundle that is positioned similarly, albeit closer toward the core of the dimer. However, when superimposing different copies of the proteins from their respective structures, the distal edge superposes relatively poorly in ChsH2 while the equivalent residues in Shy_MaoC_ superpose much more closely, suggesting that Shy_MaoC_ is relatively less flexible. This may reflect their substrate specificity, as Shy_MaoC_ only needs to interact with one face of the amphipathic bile acid substrate, while ChsH2 used a cholesterol substrate where both faces are hydrophobic. This flexibility may allow the ChsH2 active site to partially close so as to bury both faces of the substrate in a productive complex.

While intuitively the heteromeric/pseudo-heteromeric architecture seems less efficient, it allows the specialized protomer to be optimized for stability and dynamics, separately from the evolutionary pressure for activity. As demonstrated by the catalytic efficiency of ChsH1-ChsH2 and its bile acid degrading homologs, this does not result in any net loss in productivity relative to Shy_MaoC_ (25). Another potential advantage can be observed in Actinobacterial heteromeric MaoC type enzymes involved in synthesis of long-chain fatty acids that are incorporated into mycolic acids of the cell wall. In this system, HadB contains the catalytic residues while HadA and HadC do not (40). HadB can associate with either HadA or HadC, forming HadAB or HadBC complexes. Interestingly these complexes have distinct substrate specificities, with HadAB preferring C12-C18 fatty acyl chains while HadBC is specific for longer C22-C26 acyl chains (41). Crystal structures suggest that HadA and HadC bind to the acyl chains of the substrate for HadB to hydrate (40, 42). Therefore, the heteromeric architecture could enable modularity, by allowing the evolution of specialized binding partners with differing substrate specificities.

Shy’s distinct architecture from other steroid using MaoC hydratases and similarity to other proteobacterial hydratases raises interesting questions about Shy’s evolutionary origins. Previous metagenomic studies of steroid degrading bacteria have suggested that the 9,10-seco pathway for cholate A/B ring degradation originated *via* a gene duplication event of the homologous cholesterol pathway in an ancestor of *Rhodococcus* sp (43). This cluster was then horizontally transferred to a proteobacterium before subsequent further horizontal transfers between proteobacteria (43). The origin of the cholate side chain catabolism pathway in proteobacteria was, however, not explained. Based on their initial structural similarity we conducted a phylogenetic analysis of Shy, PhaJ, PaaZ, ChsH1, and ChsH2 homologs, which indicated Shy’s closest relatives are PaaZ and then PhaJ, suggesting that Shy evolved from a canonical MaoC hydratase rather than from a horizontally transferred heteromeric enzyme. Shy and ChsH1-ChsH2 therefore represent independently evolved solutions to the challenges of steroid hydration, with some convergent aspects such as the displaced active site housings and the presence of auxiliary domains, albeit with different structural compositions.

The structural and biochemical characterization of Shy sheds light on a unique steroid using enoyl-CoA hydratase. To our knowledge, Shy is the only characterized enoyl-CoA hydratase capable of using steroid substrates while maintaining a homodimeric architecture with both active sites functional. Our data are in agreement with the relationship between the size, shape, and positioning of the central helix and the upper limit of substrate size an enzyme can accept. However, many specific details on the determinants of substrate specificity in enoyl-CoA hydratases remain unclear; for example, factors determining the specificity toward steroid side chain lengths and nucleus substituents have not been elucidated. Determination of additional hydratase structures, and if possible, enzyme-substrate complexes could shed light on these details. Possible candidate for further study may be found among the anaerobic cholesterol degrading Proteobacteria, such as the aforementioned *Sterolibacterium denitrificans* (44). These enzymes could provide an avenue for comparison with bile acid utilizing Shy. This information could facilitate their use as biocatalysts to modify steroid side chains with desired steroid ring structures that can be used as synthons for producing existing or novel steroidal pharmaceuticals.

## Experimental procedures

### Chemicals

Potassium hexacyanoferrate (III) was purchased from Sigma-Aldrich. Cholic acid was purchased from Alfa Aesar. 4-pregnen-3-one-20β-carboxylic acid (3-OPC) was purchased from Steraloids Inc. Coenzyme A was purchased from BioShop Canada Inc. ATP was purchased from Bio Basic Inc. Restriction enzymes and Pfu polymerase were purchased from Thermo Fisher Scientific. T4 DNA ligase was purchased from New England Biolabs. Ni^2+-^NTA Superflow resin was purchased from Qiagen. 3-OPDC-CoA and 7-HOPC-CoA were synthesized as described previously (20). All other chemicals were purchased from Thermo Fisher Scientific or Sigma-Aldrich unless otherwise stated.

### Bacterial strains and plasmids

*C. testosteroni* KF-1 and *Thermomonospora curvata* DSM 43183 were obtained from DSMZ-German Collection of Microorganisms and Cell Cultures and *E. coli* BL21 LOBSTR from Kerafast Inc.

### DNA manipulation

DNA was purified, digested, and ligated using standard protocols. The *shy, sal, shy*_*maoC*_*,* and *shy*_*DUF35*_ genes were amplified from the genomic DNA of *C. testosteroni* KF-1 with the primers listed in Table S3. DNA fragments containing *shy, shy*_*maoC*_*,* and *shy*_*DUF35*_ were inserted between the NdeI and HindIII sites of pBTLactac plasmid that would enable expression of tagless proteins in *E. coli.* The pBTLactac plasmid was derived from pBTL-4 plasmid (45), with the weak *lac* promoter removed by BsrBI and HindIII digestion and replaced with the *lacI*^*q*^ and *tac* promoter from pVLT31 (46). The DNA fragment containing *shy*_*maoC*_ was inserted into pET28a using NdeI and HindIII to produce His-tagged protein (Novagen). DNA containing *sal* was inserted into pMCSG7 (47) *via* ligation independent cloning as there are internal NdeI sites in the gene that preclude restriction site-based insertion into common *E. coli* expression vectors. Plasmids were transformed into *E. coli* BL21 LOBSTR for expression. Site-directed mutagenesis was performed *via* a modified QuikChange method using primers listed in Table S3 (48). Cloned genes and mutations were confirmed by DNA sequencing at Laboratory Services (University of Guelph).

### Protein expression and purification

Recombinant *E. coli* was grown in 4 L of LB media supplemented with kanamycin (50 μg/ml), ampicillin (100 μg/ml), and/or tetracycline (15 μg/ml) at 37 °C. At mid-log phase (*A*_600_ of 0.4–0.6) recombinant protein expression was induced by the addition of 1 mM IPTG. Cells were incubated for a further 24 h at 15 °C and harvested by centrifugation at 9605*g* for 10 min. *E. coli* cell pellets were resuspended in 20 mM Hepes buffer (pH 7.5) and lysed by passage through a French press at 103,421 kPa. Cell lysates were centrifuged at 39,191*g* for 15 min. Cell extracts were filtered through a 0.45 μm filter and incubated for 1 h at 4 °C with Ni^2+-^NTA resin in buffer (50 mM sodium phosphate buffer pH 8.0, 300 mM sodium chloride) containing 20 mM imidazole. The mixture was poured into a gravity column and washed with the same buffer. The His-tagged proteins were eluted with buffer containing 150 mM imidazole (pH 8.0). The buffer was exchanged for 20 mM Hepes (pH 7.5) by dilution in a stirred cell equipped with a YM10 filter (Amicon). Purified enzymes were stored at −80 °C.

### Determination of protein concentrations, purities, and molecular masses

The concentration of purified proteins was determined using a Bradford assay with bovine serum albumin as a standard (49). Enzyme purity was assessed using Coomassie blue-stained SDS-PAGE gel. Gel was imaged using Bio-rad GelDoc interfaced with the software Imagelab (https://www.bio-rad.com/en-ca/product/image-lab-software?ID=KRE6P5E8Z&s_kwcid=AL%2118120%213%21657783331339%21b%21%21g%21%21image+lab+software%2120103027788%21148476196066&WT_mc_id=240108040415&WT_srch=1&WT_knsh_id=94b77e2c-1185-4e6f-b2d4-15aaf8d9556d&gad_source=1&gclid=EAIaIQobChMIx6zwxf-ehwMVhxStBh2cWQdpEAAYAiAAEgJjqfD_BwE). The native molecular weights of purified enzymes were estimated using analytical size-exclusion chromatography using a Superdex 200 column (Cytiva) with 20 mM sodium Hepes buffer pH 7.5 containing 0.15 M NaCl as the equilibration and elution buffer. The standard curve used consisted of the proteins cytochrome c (*Mr* = 12,400), carbonic anhydrase (*Mr* = 29,000), bovine serum albumin (*Mr* = 66,000), alcohol dehydrogenase (*Mr* = 150,000), and β-amylase (*Mr* = 200,000) (Sigma-Aldrich).

### CD spectroscopy

Protein solutions (0.15 mg/ml) were prepared in 1 mM sodium phosphate, pH 7.0, buffer and analyzed in 1 mm path-length quartz cuvettes. CD spectra were collected using a Jasco, Inc, J-815 spectropolarimeter from 260 to 190 nm with 0.5 nm pitch, 1 nm bandwidth, 2 s of data integration time, and 100 nm/min scan speed. Nine individual spectra were averaged and then blank subtracted, followed by processing *via* a Savitzky–Golay filter in Jasco, Inc, Spectra Manager 2 software (https://jascoinc.com/products/spectroscopy/molecular-spectroscopy-software/).

### Cholyl-enoyl-CoA and cholyl-3-OH-CoA synthesis

Cholyl-enoyl-CoA was synthesized similarly to previously described methods (25). A 10 ml reaction mixture of 100 mM sodium Hepes buffer (pH 7.5) with cholic acid (1.0 mM), CoA (1.0 mM), ATP (2.5 mM), magnesium sulfate (5.0 mM), and the acyl-CoA synthetase CasG from *Rhodococcus jostii* RHA1 (15) (1.5 μM) was incubated overnight at 22 °C with gentle agitation. Produced cholyl-CoA was dehydrogenated to cholyl-enoyl-CoA using the acyl-CoA dehydrogenase CasC from *R. jostii* RHA1 (14) (500 nM) and potassium hexacyanoferrate (III) (300 μM) in 40 ml 100 mM sodium Hepes buffer (pH 8.5) for 2.5 h at room temperature with gentle agitation. To produce cholyl-3-OH-CoA, the hydratase ChsH3 from *M. tuberculosis* (1 μM) was added (25). The reaction was then halted by acidification to pH 4.0 with HCl and the solution passed through 0.45 μm and YM10 filters. Twenty milliliters of the filtrate was loaded onto a 2.8 ml HyperSep Disposable C18 column (500 mg bed weight; Thermo Fisher Scientific) equilibrated with 10% acetonitrile in 50 mM sodium phosphate buffer (pH 5.3). The column was washed with the same buffer and CoA-esters were eluted with 10 ml 40% acetonitrile in 50 mM sodium phosphate buffer (pH 5.3). This process was repeated twice to purify all the filtrate. Acetonitrile was evaporated and water was removed by lyophilization. The identities of the steroid esters were confirmed by electrospray ionization-mass spectrometry.

### 3β-hydroxy-chol-5,22-diene-24-oyl-CoA (3-HCDO-CoA) synthesis

3β-hydroxy-Δ^5^-cholenoic acid was CoA-esterified to 3β-hydroxy-5-cholen-24-oyl-CoA *via* a mixed anhydride reaction due to limited water solubility of the parent compounds as previously described (50). Briefly, 3β-hydroxy-Δ^5^-cholenoic acid (0.13 mmole) and triethylamine (0.26 mmole) were dissolved in 6 ml dichloromethane. A solution of ethyl chloroformate (0.26 mmole) in 2 ml dichloromethane was added to the dissolved steroid acid on ice and shaken occasionally at room temperature for 2 h. The dichloromethane was evaporated, and the produced anhydride was dissolved in 5 ml tetrahydrofuran. CoA (0.06 mmoles) in 5 ml water adjusted to pH 8.0 was added to the mixed anhydride. The solution was acidified to pH 5.0 with 1% perchloric acid and tetrahydrofuran was removed by evaporation. Thioesters were precipitated by addition of 1 ml 10% perchloric acid and collected by centrifugation followed by washing with 1 ml ethyl ether. Produced 3β-hydroxy-5-cholen-24-oyl-CoA was dehydrogenated to 3-HCDO-CoA using the acyl-CoA dehydrogenase CasC from *R. jostii* RHA1 (14) (500 nM) and potassium hexacyanoferrate (III) (300 μM) in 40 ml 100 mM sodium Hepes buffer (pH 8.5) for 2.5 h at room temperature with gentle agitation (24). Produced 3-HCDO-CoA was purified by the same procedure used for cholyl-enoyl-CoA; however, the equilibration/wash and elution buffers contained 20 and 80% acetonitrile, respectively. The identity of the produced steroid enoyl-CoA ester was confirmed by electrospray ionization-mass spectrometry.

### Analysis of CoA esters and reaction products

Reactions were performed with 50 μM cholyl-enoyl-CoA or cholyl-3-OH-CoA in 100 mM Hepes pH 7.5 buffer. CoA-ester substrates and products were analyzed on an Agilent 1200 HPLC liquid chromatograph interfaced with an Agilent UHD 6530 Q-Tof mass spectrometer at the Mass Spectrometry Facility of the Advanced Analysis Centre, University of Guelph. A C18 column (Agilent Poroshell 120, 50 mm × 4.6 mm 2.7 μm) was used for chromatographic separation of 10 μl sample volume with the following solvents: water with 0.1% formic acid for A and acetonitrile with 0.1% formic acid for B. The mobile phase gradient was as follows: initial conditions, 5% B increasing to 100% B in 15 min followed by column wash at 100% B and 10 min reequilibration. The flow rate was maintained at 0.4 ml/min. The mass spectrometer electrospray capillary voltage was maintained at 4.0 kV and the drying gas temperature at 250 °C with a flow rate of 8 L/min. Nebulizer pressure was 30 psi, and the fragmentor was set to 160. Nitrogen was used as nebulizing gas, drying gas, and collision-induced dissociation gas. The mass-to-charge ratio was scanned across the m/z range of 50 to 2000 m/z in 4 GHz (extended dynamic range positive- and negative-ion auto MS/MS modes). Two precursor ions per cycle were selected for MS2 fragmentation scanning from 25 to 2000 m/z. The instrument was externally calibrated with the ESI Tuning Mix (Agilent Technologies). The data were analyzed using Agilent Qualitative Analysis software 10 (https://www.agilent.com/en/product/software-informatics/mass-spectrometry-software/data-analysis/qualitative-analysis).

### Steady-state kinetic assays

All assays were performed in at least triplicate, in 100 mM sodium Hepes buffer (pH 7.5) with a total volume of 1 ml, at 25 °C, using a Varian Cary 3 spectrophotometer equipped with a temperature-controlled cuvette holder. Hydratase activity was determined by measuring the decrease in absorbance at 263 nm due to Cα-Cβ saturation during hydration (ε263 of 6700 M^−1^ cm^−1^) (Fig. S10) (51). Aldolase activity against cholyl-3-OH-CoA was performed with 1 mM NAD^+^ and 7 nM *C. testosteroni* KF-1 Sad. Activity was determined by measuring increase in absorbance at 340 nm corresponding to reduction of NAD^+^ to NADH (ε340 of 6220 M^−1^ cm^−1^). Data were fitted to the Michaelis–Menten equation by nonlinear regression using GraphPad Prism software (https://www.graphpad.com/).

### Protein crystallization and structure determination

Conditions for crystallization of Shy_MaoC_ were screened using the JCSG+ and PACT premier kits (Molecular Dimensions Inc). Crystals used for data collection were grown using the sitting drop method at 4 °C, with 1 μl of reservoir solution (0.1 M sodium cacodylate, 0.2 M calcium acetate hydrate, 40% PEG 300) combined with 1 μl 30 mM acetyl-CoA and 1 μl of 20 mg/ml Shy_MaoC_. Crystals were soaked in Paratone N as cryoprotectant prior to freezing. The datasets were collected at the Canadian Light Source, Canadian Macromolecular Crystallography Facility (CMCF-ID), and processed using XDS (52). The structure was phased using molecular replacement in Phaser, using a Colabfold model of the shy monomer as the molecular replacement model (53); Refinements were carried out in PHENIX refine (https://phenix-online.org) (54), with manual building in Coot (https://www2.mrc-lmb.cam.ac.uk/personal/pemsley/coot/) (55). Initial refinements were carried out to 1.9 Å, with the resolution cut to 2.05 Å on the final round of refinement. Figures of protein structure were generated using PyMOL version 2.0 (https://pymol.org).

### Modeling

The Shy_MaoC_ complex with cholyl-22-(*R*)-hydroxy-24-methylthioate was initially modeled by hand in Pymol, with the positioning of the substrate guided by the shape of the pocket and the interactions usually observed between the substrate and catalytic residues in productive hydratase complexes. Candidate complexes were then subjected to energy minimization in Rosetta (36).

### Sequence analysis

Multiple sequence alignment was performed using ClustalΩ (56). A maximum likelihood tree was constructed using IQ-tree with 1000 bootstrap replications (57). Sequences were selected from an NCBI BLAST search using Shy_Ctest_ and the proteins identified *via* DALI search as queries, and gene synteny was confirmed by analysis of the corresponding genome sequences in the NCBI nucleotide database.

## Data availability

Structure of Shy_MaoC_ has been deposited to the Protein Data Bank under PDB ID 8VWR. Raw diffraction data have been deposited to Zenodo at 10.5281/zenodo.11406128.

## Supporting information

This article contains supporting information (37, 39).

## Conflict of interest

MS Kimber is an editorial board member of the Journal of Biological Chemistry and was not involved in the editorial review of this manuscript. The other authors declare that they have no conflicts of interest with the contents of this article.
