# Supplementary material for: Shy is a proteobacterial steroid hydratase which catalyzes steroid side chain degradation without requiring a catalytically inert partner domain
Source: J Biol Chem. 2024 Jun 27;300(8):107509. doi: 10.1016/j.jbc.2024.107509 (PMC11321319; doi:10.1016/j.jbc.2024.107509)
Supplement: Supporting Information [file mmc1.docx]

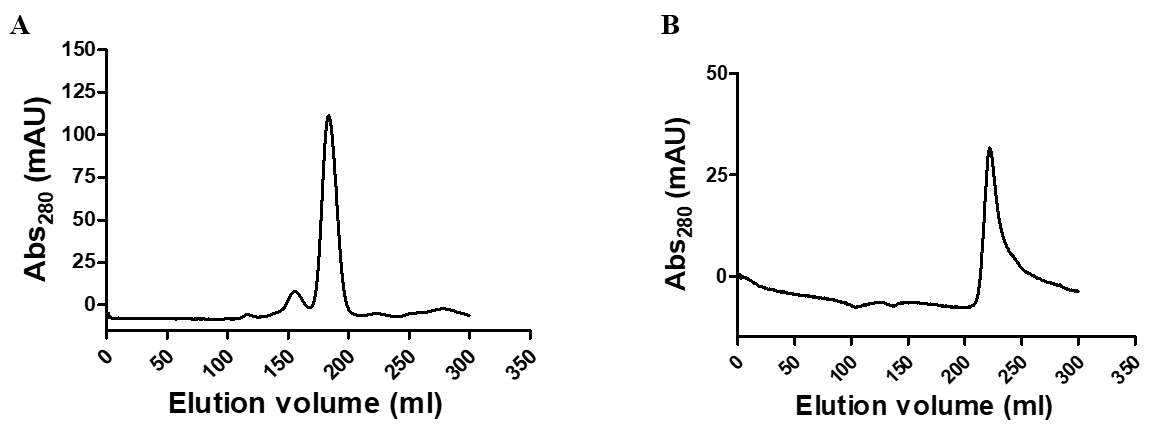


**Figure S1: Analytical size exclusion chromatographs of A) Shy-Sal and B) Shy_MaoC_**. Each measurement was performed with one replicate.

**
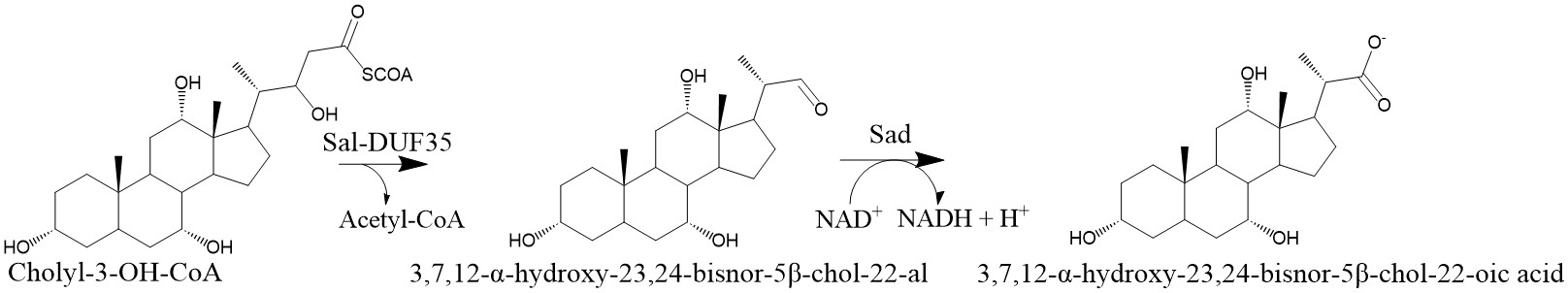
**

Figure S2: Overview of Sad coupled assay for Sal retroaldol activity. Retro aldol cleavage of Cholyl-3-OH-CoA by Sal was assayed via coupling the production of 3,7,12-α-hydroxy-23,24-bisnor-5β-chol-22-al to the reduction of NAD^+^ by Sad, which could be detected spectrophotometrically via increase in A_340_.

**
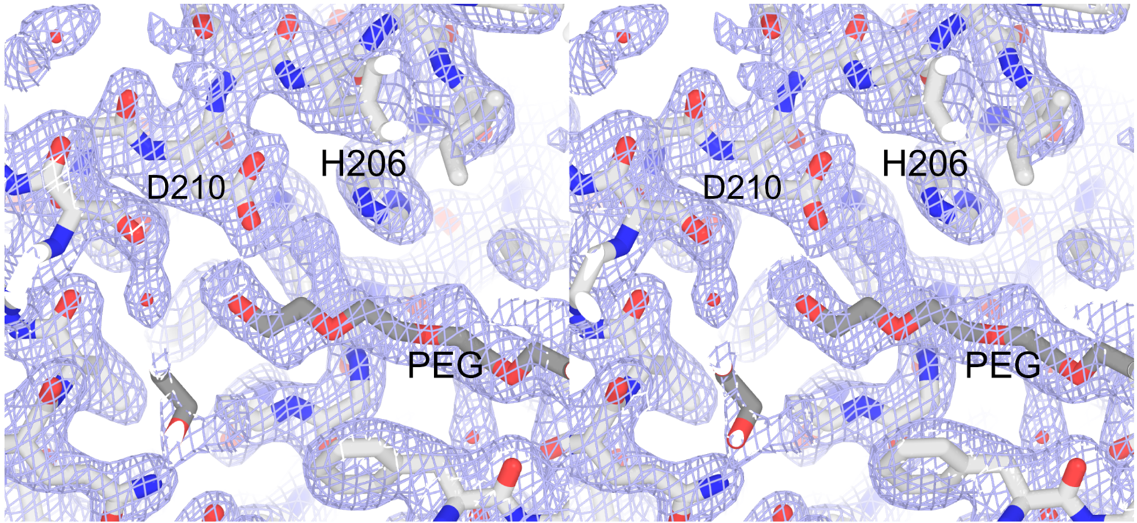
**

**Figure S3: Wall-eyed stereo diagram depicting a composite simulated anneal omit map.** The map, in blue, is contoured at 1.0 σ. The key active site residues are labelled.


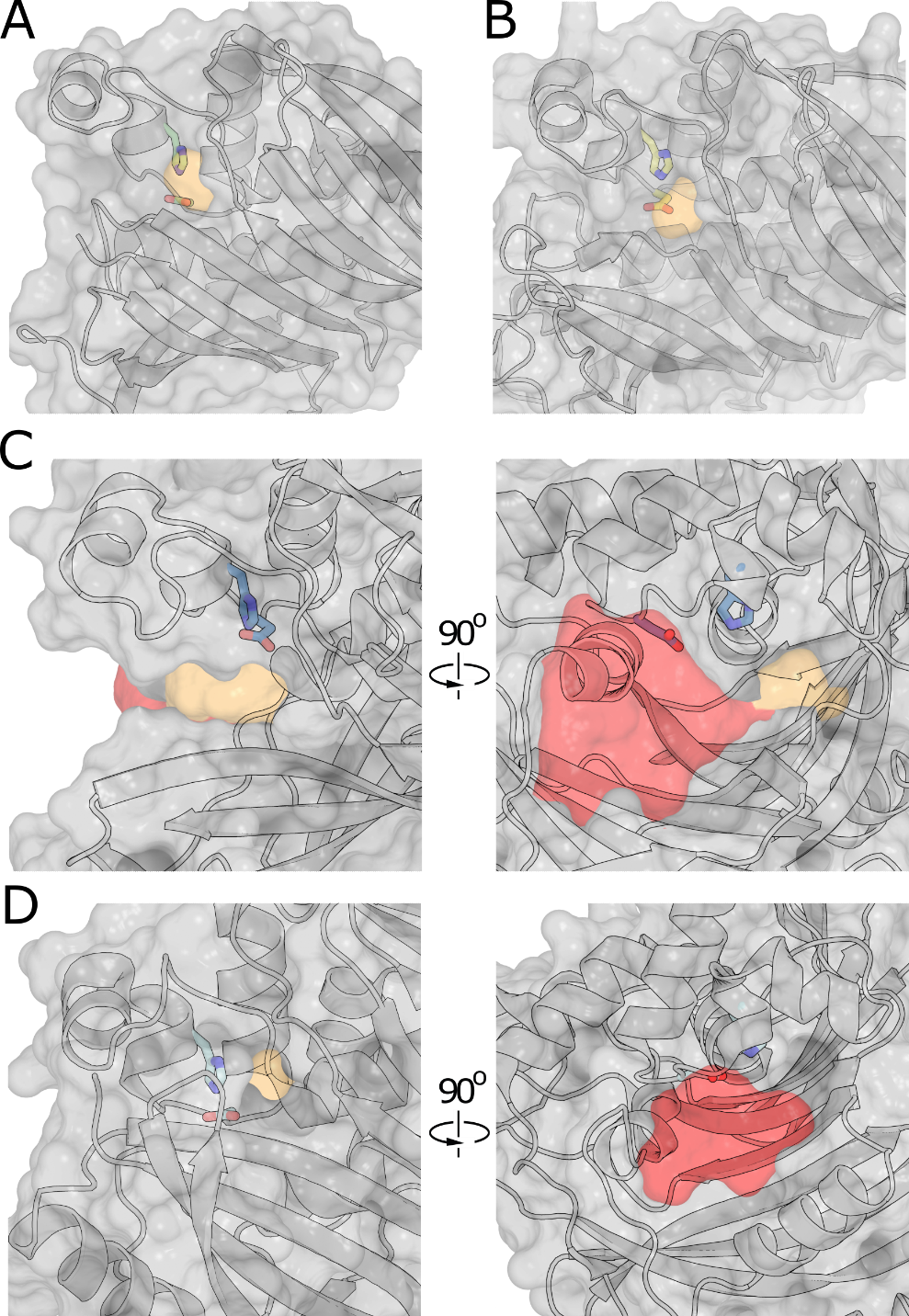


**Figure S4: Comparison of (A) PhaJ_Acav_, (B) PaaZ_MaoC_, (C) Shy_MaoC_, and (D) ChsH1-ChsH2 active site openings.** The surface of the protein is shown in grey, and the exposed volume of the active site tunnel is shown in light orange, while the exposed cleft portion of Shy and ChsH1-ChsH2 is shown in red. The catalytic dyad is shown as sticks. Pockets were detected using HOLLOW.


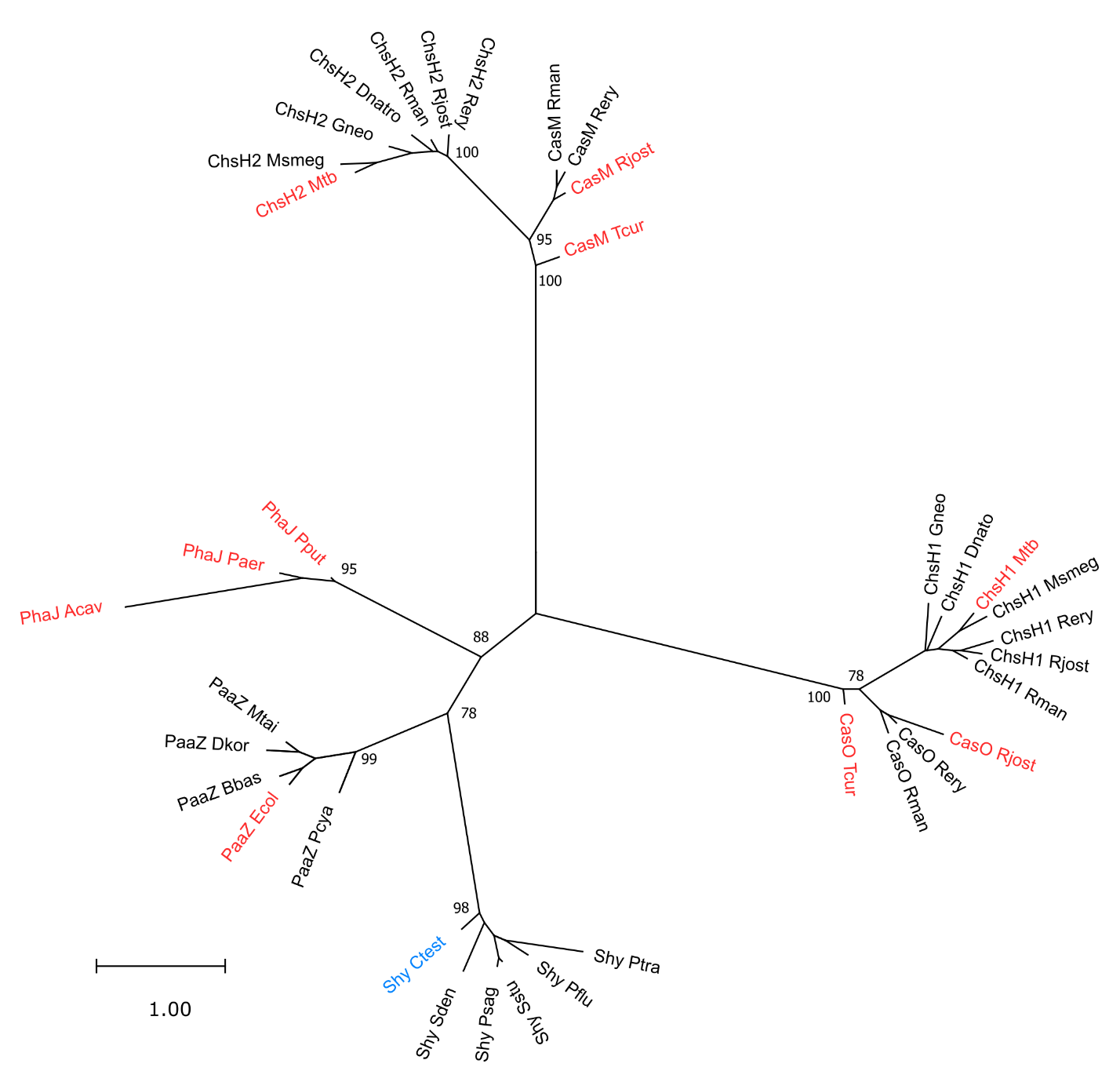


Figure S5: Unrooted maximum likelihood phylogenetic tree of Shy, PaaZ, PhaJ, and Actinobacterial heteromeric hydratases. The sources of proteins in this analysis and their sequence accession numbers are listed in Table S2. The DUF35 domains of Shy, CasM, and ChsH2 and the aldehyde dehydrogenase domains of PaaZ were removed from the alignment. Bootstrap values are displayed next to major nodes. Representative proteins that have been previously characterized are highlighted in red, while the protein from the present study is highlighted in blue.


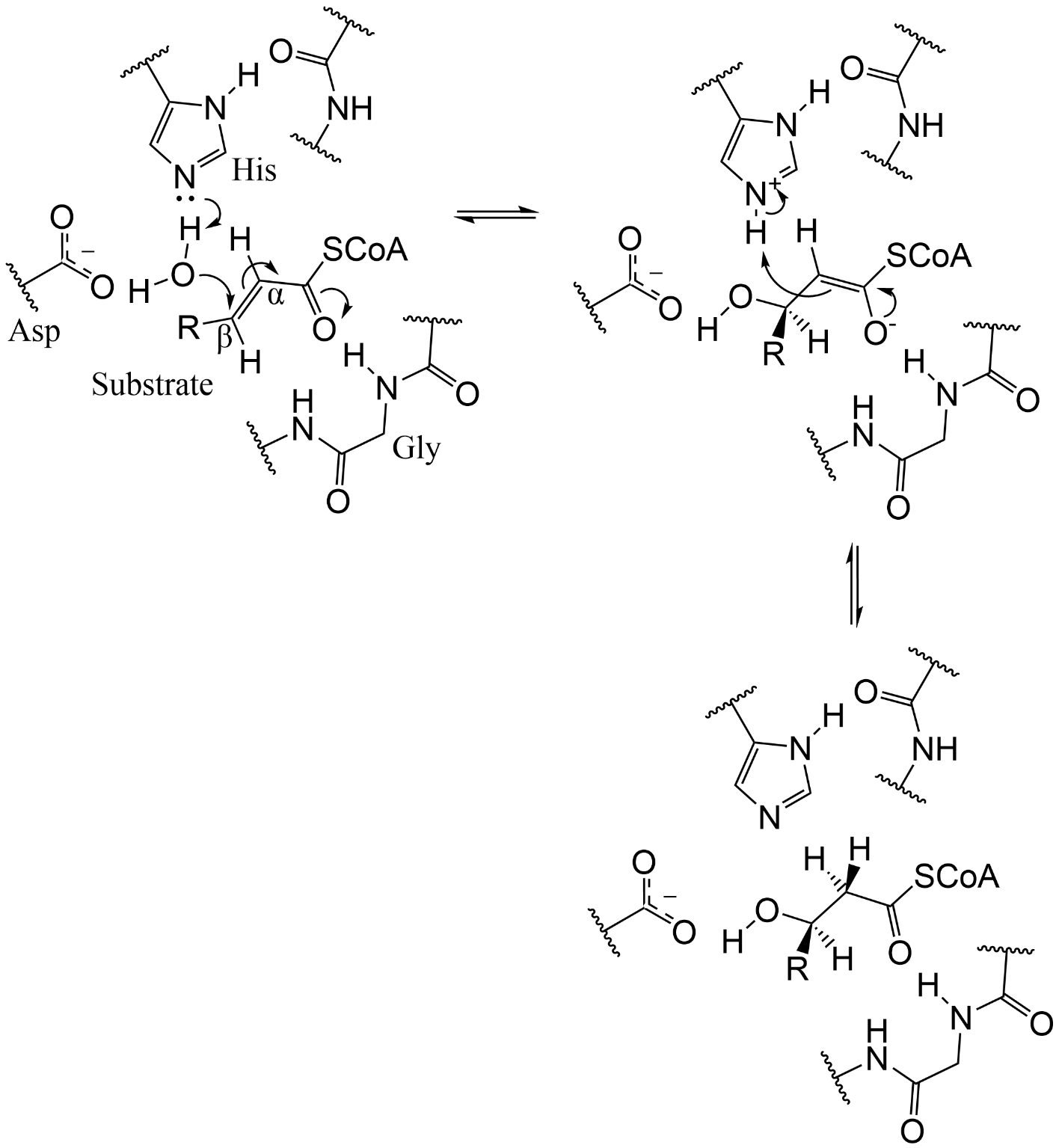


Figure S6: Reaction mechanism of MaoC enoyl-CoA hydratases.7


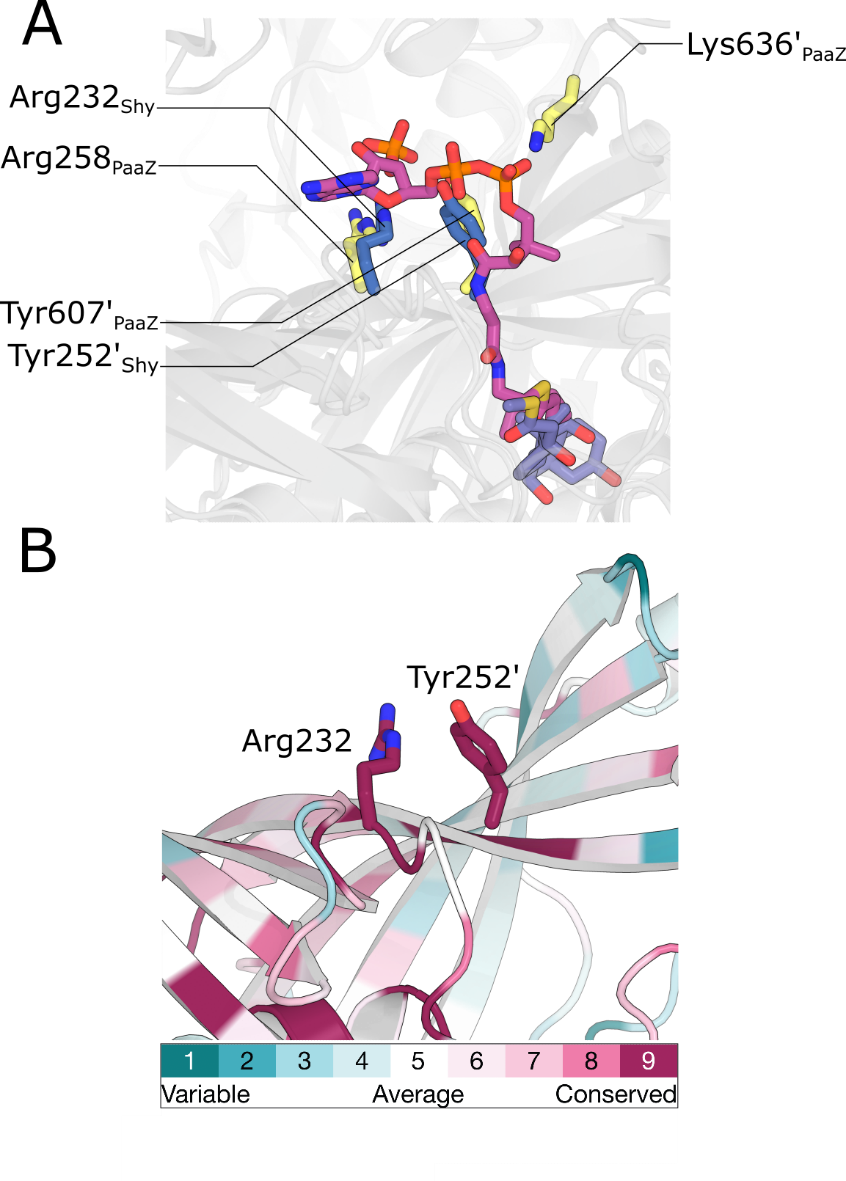


Figure S7: Potential CoA binding mode of Shy_MaoC_. (A) Structure of Shy_MaoC_ docked cholyl-22-(*R*)-hydroxy-24-methylthioate (purple sticks) superimposed with PaaZ in complex with octanoyl-CoA (magenta sticks). Residues interacting with CoA in PaaZ are shown as pale-yellow sticks while Shy residues corresponding to them are shown in blue sticks. (B) ConSurf analysis of Shy_MaoC_. Residues are color coded according to degree of conservation indicated in the scale below, with relevant residues from A shown as sticks. Residues are labeled and numbered (´ indicates residues from the opposite protomer).


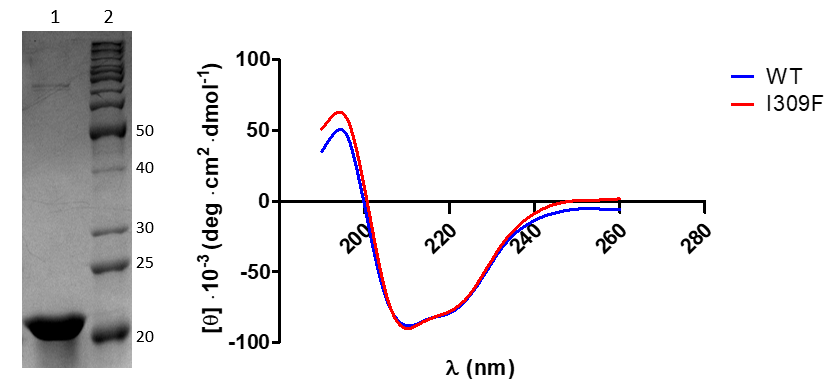


**Figure S8: SDS-PAGE gel and CD spectra of Shy_MaoC_I309F.** The protein sample is loaded in lane 1 and the molecular weight ladder is loaded in lane 2. Molecular weight in kDa of the ladder are indicated to the right. Wild type Shy_MaoC_ and I309F solutions for CD analysis were 0.15 mg/ml in 1 mM sodium phosphate, pH 7.0.


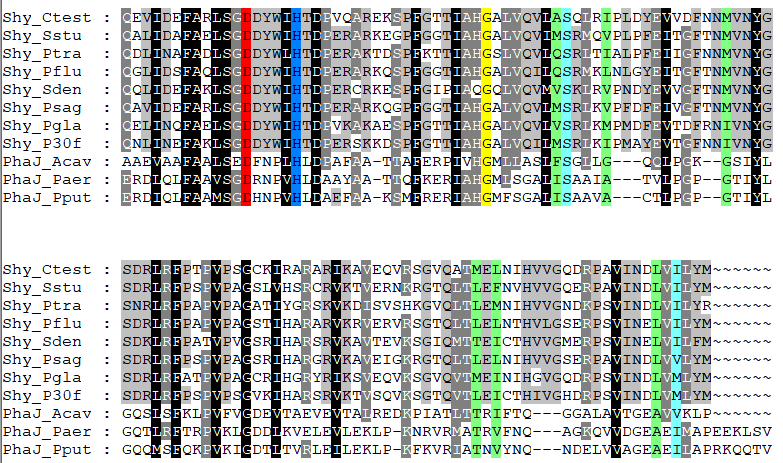


Figure S9: Section of multiple sequence alignment of Proteobacterial Shy and PhaJ homologues. The sources of proteins in this analysis and their sequence accession numbers are listed in Table S2. Shy sequences include those from bacteria experimentally confirmed to catabolize steroids used in figure S4 and sequences from unconfirmed steroid degraders that have lower sequence identity to Shy from *C. testosteroni*. The catalytic histidine and aspartate are highlighted in blue and red, respectively, while the oxyanion hole glycine is highlighted yellow. Positions shown to contribute to the substrate binding site of the PhaJs are highlighted in Cyan (37, 39). Positions of residues with side chains that define the cholyl-22-(*R*)-hydroxy-24-methylthioate nucleus binding site in the *C. testosteroni* Shy model are highlighted in green.


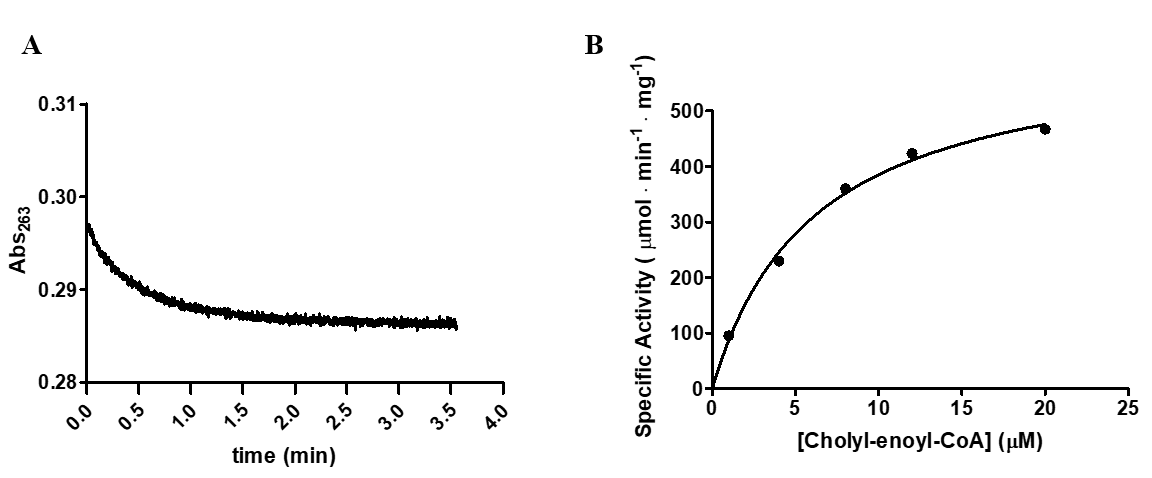


Figure S10: Michaelis-Menten plot for Shy_MaoC_ catalyzed hydration of cholyl-enoyl-CoA. Each specific activity measurement was performed at least in triplicates.

**Table S1: DALI structural alignment statistics with Shy_MaoC_.** R.m.s.d is calculated using Cα atoms

| Protein | Sequence identity (%) | r.m.s.d (Å) | Z-score |
| --- | --- | --- | --- |
| PaaZ_MaoC_ | 26 | 2.2 | 14.0 |
| PhaJ_Acav_ | 26 | 2.4 | 13.3 |
| ChsH1 | 19 | 2.5 | 12.8 |
| ChsH2 | 19 | 2.5 | 12.5 |

**Table S2: enoyl-CoA hydratase sequences used in dendrogram and multiple sequence alignment.** *^a^*indicates sequences used in multiple sequence alignment in figure S8.

| **Hydratase** | **Organism** | **Abbreviated name on tree** | **Refseq accession number** |
| --- | --- | --- | --- |
| Shy | *^a^Comamonas testosteroni* KF-1 | Ctest | WP_003057309.1 |
|  | *^a^Stutzerimonas stutzeri* | Sstu | WP_256014768.1 |
|  | *^a^****Pseudoalteromonas* *translucida*** | Ptra | WP_058372857.1 |
|  | ^a^Pseudomonas fluorescens | Pflu | WP_095020036.1 |
|  | *^a^****Pseudomonas* *sagittaria*** | Psag | WP_092429282.1 |
|  | ^a^Sterolibacterium denitrificans | Sden | WP_154716410.1 |
|  | *^a^Polaromonas glacialis* | N/A | WP_029527744.1 |
|  | *^a^Polynucleobacter* sp. 30F-ANTBAC | N/A | WP_216287656.1 |
| PhaJ | *^a^Aeromonas caviae* | Acav | WP_168235189.1 |
|  | *^a^Pseudomonas aeruginosa* | Paer | WP_003091647.1 |
|  | *^a^Pseudomonas putida* | Pput | [WP_046816629.1](https://www.ncbi.nlm.nih.gov/protein/WP_046816629.1?report=genbank&log$=prottop&blast_rank=1&RID=CKMCCZFA01N) |
| PaaZ | *Escherichia coli* K-1 | Ecol | WP_001186469.1 |
|  | *Deinococcus koreensis* | Dkor | WP_103312003.1 |
|  | *Beauveria bassiana* D1-5 | Bbas | KGQ06108.1 |
|  | *Meiothermus taiwanensis* WR-220 | Mtai | AWR86696.1 |
|  | *Pleurocapsales cyanobacterium* LEGE 10410 | Pcya | MBE9048094.1 |
| ChsH1 | *Mycobacterium tuberculosis* | Mtb | WP_003419290.1 |
|  | *Mycolicibacterium smegmatis* | Msmeg | **WP_174519625.1** |
|  | *Gorrdonia neofelifaecis* | Gneo | **WP_009680550.1** |
|  | *Dietzia natronolimnaea* | Dnato | **WP_017836006.1** |
|  | *Rhodococcus erythropolis* PR4 | Rery | **WP_020968404.1** |
|  | *Rhodococcus jostii* RHA1 | Rjost | **WP_011596877.1** |
|  | *Rhodococcus maanshanensis* | Rman | **WP_077042936.1** |
| ChsH2 | *Mycobacterium tuberculosis* H37Rv | Mtb | **WP_003419293.1** |
|  | *Mycolicibacterium smegmatis* | Msmeg | **WP_253801979.1** |
|  | *Gorrdonia neofelifaecis* | Gneo | **WP_009680551.1** |
|  | *Dietzia natronolimnaea* | Dnato | **WP_095719052.1** |
|  | *Rhodococcus erythropolis* PR4 | Rery | **WP_011596876.1** |
|  | *Rhodococcus jostii* RHA1 | Rjost | **WP_011596876.1** |
|  | *Rhodococcus maanshanensis* | Rman | **WP_097214262.1** |
| CasM | *Rhodococcus erythropolis* PR4 | Rery | WP_269576882.1 |
|  | *Rhodococcus jostii* RHA1 | Rjost | WP_011597930.1 |
|  | *Rhodococcus maanshanensis* | Rman | WP_072750640.1 |
|  | *Thermomonospora curvata* DSM 43183 | Tcur | WP_012853804.1 |
| CasO | *Rhodococcus erythropolis* PR4 | Rery | WP_020909133.1 |
|  | *Rhodococcus jostii* RHA1 | Rjost | WP_011597932.1 |
|  | *Rhodococcus maanshanensis* | Rman | WP_072750642.1 |
|  | *Thermomonospora curvata* DSM 43183 | Tcur | WP_012853806.1 |

**Table S3: Sequence of primers used to PCR amplify *shy* and *sal* genes.** Underlined sequences indicate introduced restriction sites while bold sequences indicate mutated codons.

| Primer | Primer sequence |
| --- | --- |
| *shy* | GCCGCATATGGAGTCCGAGGTTGCCGTAGTG  GCGCAAGCTTCACATGTAGAGAATCACCAGG |
| *sal* | TACTTCCAATCCAATGCCATGGGTTTGCAAGGAAAAGCGGCGCTGG  TTATCCACTTCCAATGTTATGCTCCCCGCAGGATCAAGGC |
| *shy_MaoC_* | CATATGGAGTCCGAGGTTGCCGTAGTG  GCGCAAGCTTCACATGTAGAGAATCACCAGG |
| *Shy_DUF35_* | CGCGCATATGGCTTGGAACAAACCTCTGC  GCGCAAGCTTCAATCGGCCTTGATGACGCTCG |
| *shy_MaoC_I309F* | CGACCTGGTG **TTT** CTCTACATGTGAAGCTTGCGGCCGCAC  CATGTAGAG**AAA**CACCAGGTCGTTGATGACGGCCGGACGATCC |
| *shy_MaoC_L307F* | CATCAACGAC**TTC**GTGATTCTCTACATGTGAAGCTTGCGGCCG  AGAGAATCAC**GAA**GTCGTTGATGACGGCCGGACGATCCTG |
| *shy_MaoC_H206Q* | TACTGGATT**CAG**ACCGACCCCGTGCAGGCGCGTGAGAAAAG  GGGTCGGT**CTG**AATCCAGTAGTCATCGCCCGAGAGCCTG |
| *shy_MaoC_H206A* | TACTGGATT**GCC**ACCGACCCCGTGCAGGCGCGTGAGAAAAG  GGTCGGT**GGC**AATCCAGTAGTCATCGCCCGAGAGCCTGG |
